# Exploring the role of interdisciplinarity in physics: Success, talent and luck

**DOI:** 10.1371/journal.pone.0218793

**Published:** 2019-06-26

**Authors:** Alessandro Pluchino, Giulio Burgio, Andrea Rapisarda, Alessio Emanuele Biondo, Alfredo Pulvirenti, Alfredo Ferro, Toni Giorgino

**Affiliations:** 1 Department of Physics and Astronomy “Ettore Majorana”, University of Catania, Catania, Italy; 2 INFN Sezione di Catania, Catania, Italy; 3 Complexity Science Hub Vienna, Austria; 4 Department of Economics and Business, University of Catania, Catania, Italy; 5 Department of Clinical and Experimental Medicine, University of Catania, Catania, Italy; 6 Institute of Biophysics, National Research Council of Italy, Milan, Italy; 7 Department of Biosciences, University of Milan, Milan, Italy; Universidad Veracruzana, MEXICO

## Abstract

Although interdisciplinarity is often touted as a necessity for modern research, the evidence on the relative impact of sectorial versus to interdisciplinary science is qualitative at best. In this paper we leverage the bibliographic data set of the American Physical Society to quantify the role of interdisciplinarity in physics, and that of talent and luck in achieving success in scientific careers. We analyze a period of 30 years (1980-2009) tagging papers and their authors by means of the Physics and Astronomy Classification Scheme (PACS), to show that some degree of interdisciplinarity is quite helpful to reach success, measured as a proxy of either the number of articles or the citations score. We also propose an agent-based model of the publication-reputation-citation dynamics which reproduces the trends observed in the APS data set. On the one hand, the results highlight the crucial role of randomness and serendipity in real scientific research; on the other, they shed light on a counterintuitive effect indicating that the most talented authors are not necessarily the most successful ones.

## Introduction

The importance and the beneficial role of interdisciplinarity is very often advocated in editorials of high impact journals, public speeches about innovative research policies and research proposal guidelines [1–5]. However, starting and pursuing interdisciplinarity research projects is fraught with difficulties and risks. First of all, funds are difficult to obtain because the evaluation of projects is frequently underestimated or misjudged, since they pertain to different disciplines, thus requiring a much broader assessment. Secondly, interdisciplinary groups are somehow risky for young researchers, because a hybrid curriculum does not help in getting career advancements. Thirdly, developing a common language among scientists with different backgrounds is tough and very time demanding.

Despite such difficulties, there are several indications that the interdisciplinary character of research is growing and that this can be considered a positive signal for the progress of science [6, 7]. In the last decades we have seen the birth of new disciplines originated by the contamination of physics with biology, computer science and big data, finance, economics, and social sciences in general [8–11]. Most of this hybridization is due to the acknowledgement of the complex nature of socio-economic phenomena [12], which in turn fostered research on the field of complex systems in universities and research centers, attracting scientists coming from different backgrounds. In this context, physicists and economists have recently tried to understand the determinants of innovation and success [6, 13–21] from a new and rigorous perspective. In this study we investigate the role of interdisciplinarity in physics research and question how much it is a key ingredient for a successful career. In particular, we address the publication-reputation-citation dynamics of the physicists research community by exploiting the information extracted from the American Physical Society (APS) data set. In the first part of the paper, we evaluate how much the individual propensity to interdisciplinarity influences the score of an author in terms of both publications and citations. In the second part, inspired by a recent study [19], we present an agent-based model that quantitatively reproduces the stylized facts of empirical APS data. In this context, in agreement with a research line aimed to show the beneficial role of randomness in social and economic systems [22–26], we also try to analyse the role of chance in reaching high levels of success in scientific careers.

## APS data set: Materials and methods

The APS data set is a corpus of articles published in Physical Review Letters, Physical Review and Reviews of Modern Physics, and dates back to 1893 (see Appendix 1 in the Supporting Information for more details about this section). In particular, data about of all citing-cited pairs of articles in which one paper cites another within the collection, basic metadata and sub-disciplinary classifications about each article in the collection, are present. Data in APS data set were preprocessed and cleaned to avoid duplicated authors and affiliations by using Jaccard similarity in connection to Locality Sensitive Hashing. For the aims of our analysis we will only consider the period 1980–2009 and the articles of the *N* = 7303 authors who published their first paper between 1975 and 1985 and at least three papers between 1980 to 2009. Their total scientific production in this interval of 30 years consists of 89949 PACS classified articles, which received a total of 1329374 citations from the other articles in the data set. The Physics and Astronomy Classification Scheme (PACS) is a hierarchical partitioning of the whole spectrum of subject matter in physics, astronomy, and related sciences introduced since 1975. We limit our attention to the 10 most general PACS classes, each corresponding to a broad disciplinary field. Information about classes present in each article are available in the APS data set.

For a given author *A*_*i*_ (*i* = 1, …, *N*), the total number $D_{i}^{APS}\in \left[1,10\right]$ of different PACS classes appearing in her publications during her entire career could be certainly considered as a global indicator of the multidisciplinarity of her work. However, as the $D_{i}^{APS}$ classes do not appear simultaneously in all the papers of *A*_*i*_, it is also interesting to consider the average number $d_{i}^{APS}\in R$ of classes simultaneously present in her articles. From the APS data it results that $d_{i}^{APS}\in \left[1,3.33\right]$. Multiplying these two factors, we finally obtain the index

$$\begin{array}{c} I_{i}^{APS}=D_{i}^{APS}\times d_{i}^{APS} \end{array}$$ (1)

which we propose as a more robust indicator of interdisciplinarity.

Accordingly, we can divide the *N* = 7303 authors into three groups, with comparable sizes and with an increasing level of interdisciplinarity:

- Level 1 group $L_{1}^{APS}$: *N*_1_ = 2445 authors with $1\leq I_{i}^{APS}\leq 3$ (low interdisciplinarity level);
- Level 2 group $L_{2}^{APS}$: *N*_2_ = 2511 authors with $3<I_{i}^{APS}\leq 6$ (medium interdisciplinarity level);
- Level 3 group $L_{3}^{APS}$: *N*_3_ = 2347 authors with $I_{i}^{APS}>6$ (high interdisciplinarity level);

The first goal of this study is to investigate if these different degrees of interdisciplinarity are correlated to the scientific impact of the active researchers of the APS data set, evaluated through both the number of papers and the citations cumulated during their careers.

## Results

**Fig 1:**
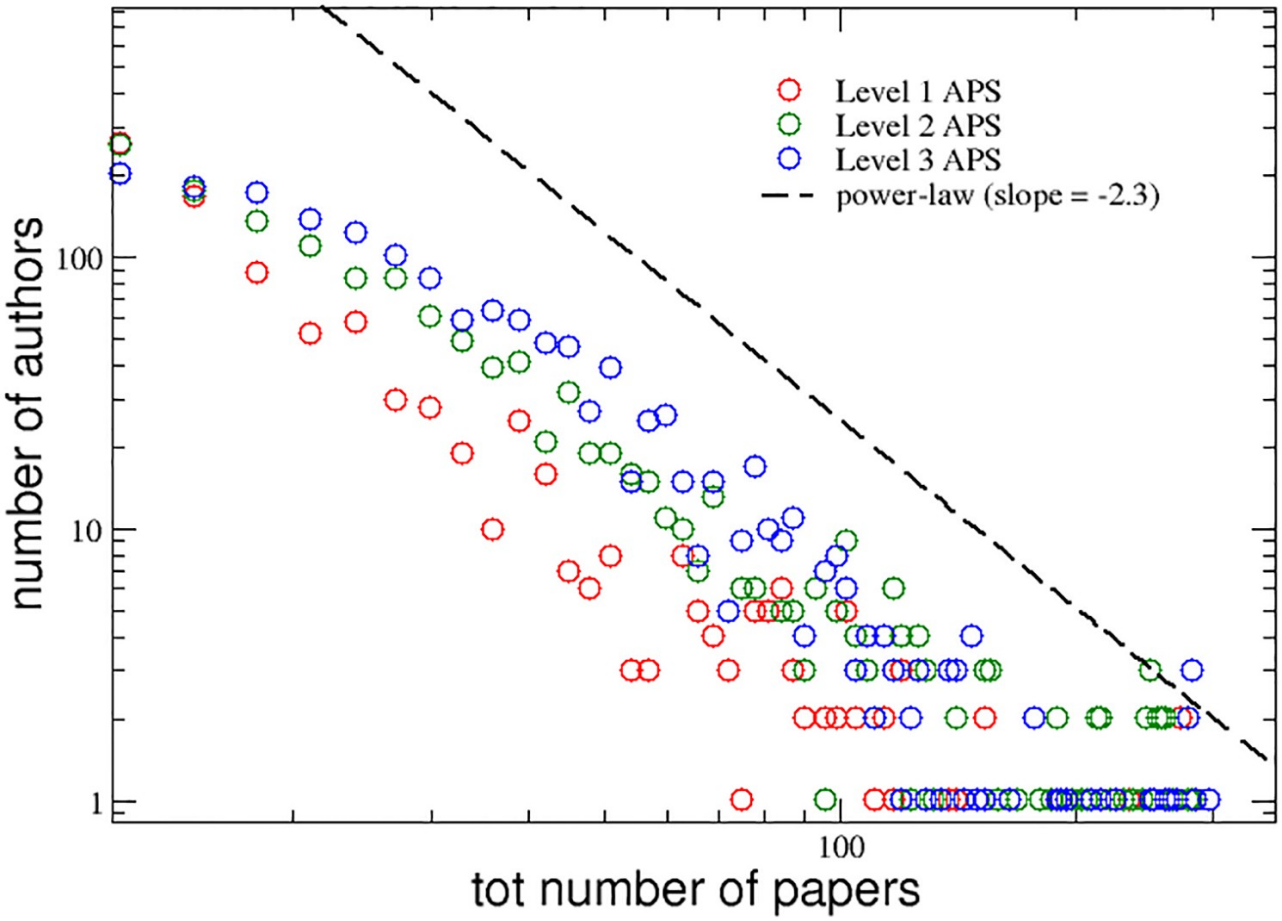
APS data set. Distributions of the total number of papers published, during their entire careers, by the authors of the three groups with increasing levels of interdisciplinarity. A power-law curve with slope equal to −2.3 is also reported for comparison (dashed line).

Fig 1 shows the distributions of the total number of papers published by the authors belonging to the three groups $L_{1}^{APS}$, $L_{2}^{APS}$ and $L_{3}^{APS}$, which are plotted in three different colors (red, green and blue, respectively). The interdisciplinarity level has a strong positive influence on the productivity of the authors, since authors that have a higher level of interdisciplinarity are more productive. In addition, the tails of the three distributions follow a power-law behavior, with a slope equal to −2.3 (dashed line).

**Fig 2:**
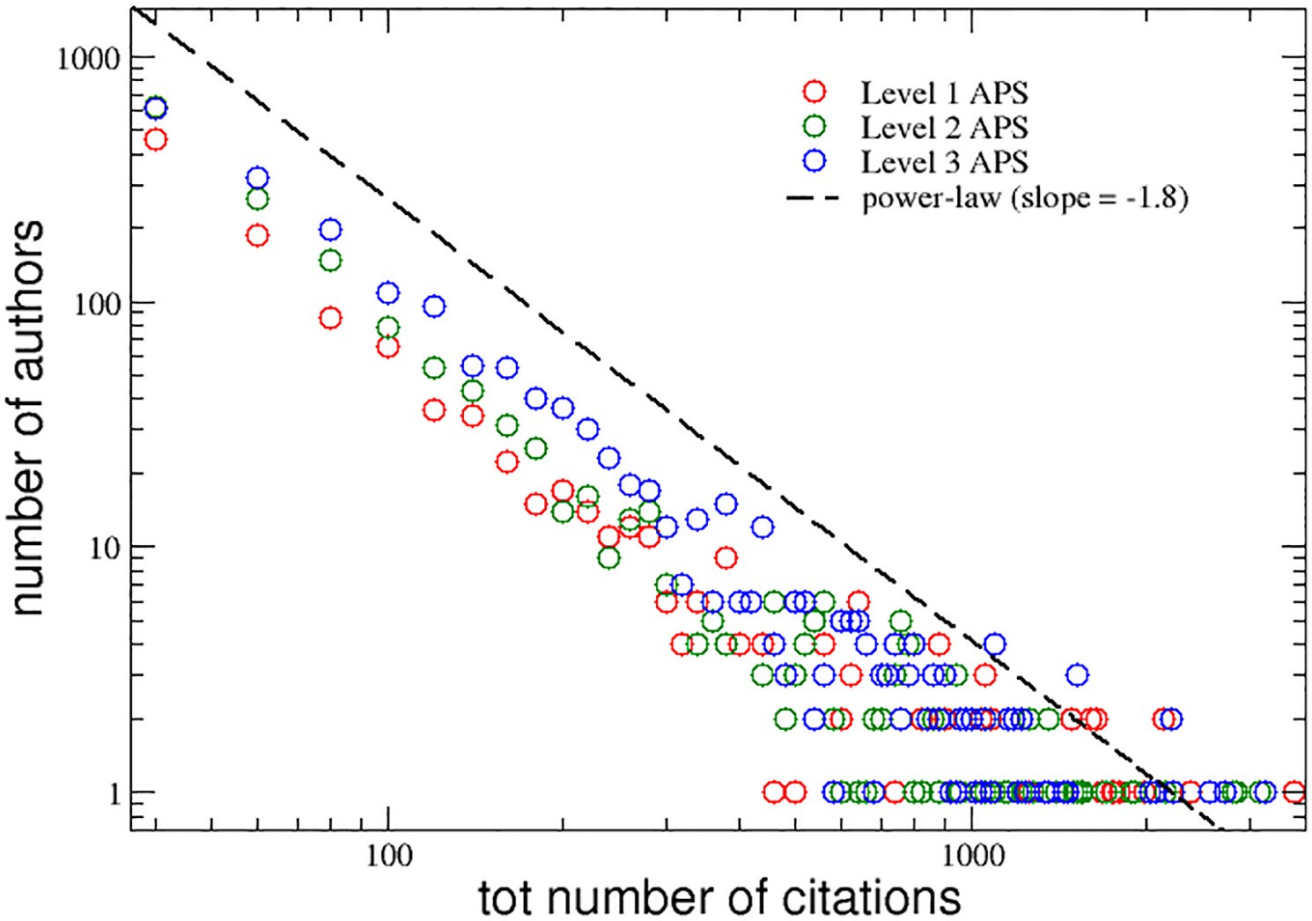
APS data set. Distributions of the total number of citations cumulated, during their entire careers, by the authors of the three groups with increasing levels of interdisciplinarity. A power-law curve with slope -1.8 is also reported for comparison (dashed line).

A similar behavior is visible in Fig 2, where the distributions of the total number of citations received by the authors of the three groups during their entire careers is plotted with the same colors. Also in this case, the interdisciplinarity level seems to play an important role in affecting the scientific success of the researchers. Again, the tails of the three distributions follow a power-law behavior, but here with a different slope equal to −1.8.

In Table 1 we report the total number of papers and citations for each interdisciplinarity group, together with the corresponding averages per author ($\bar{P}$ and $\bar{C}$, respectively). The results confirm the hypothesis that $I_{i}^{APS}$ is able to capture a real interesting effect encoded in the APS data set, i.e. the beneficial role of interdisciplinarity in enhancing both the productivity and the scientific impact of the examined authors.

**Table 1:** Some characteristic numbers of our APS sample regarding the 89949 published papers and their 1329374 citations over the three interdisciplinarity groups. Here ${\bar{P}}^{APS}$ = average papers per author and ${\bar{C}}^{APS}$ = average citations per author. A paper is counted in more than one class if it is coauthored by researchers belonging to different classes, so the sums of the number of papers and of the citations exceed, respectively, 89949 and 1329374.

	Authors	Papers	${\bar{P}}^{APS}$	Citations	${\bar{C}}^{APS}$
$L_{1}^{APS}$	2445	18832	7.70	230448	94.25
$L_{2}^{APS}$	2511	35892	14.29	515635	205.35
$L_{3}^{APS}$	2347	50947	21.71	843292	359.31

**Fig 3:**
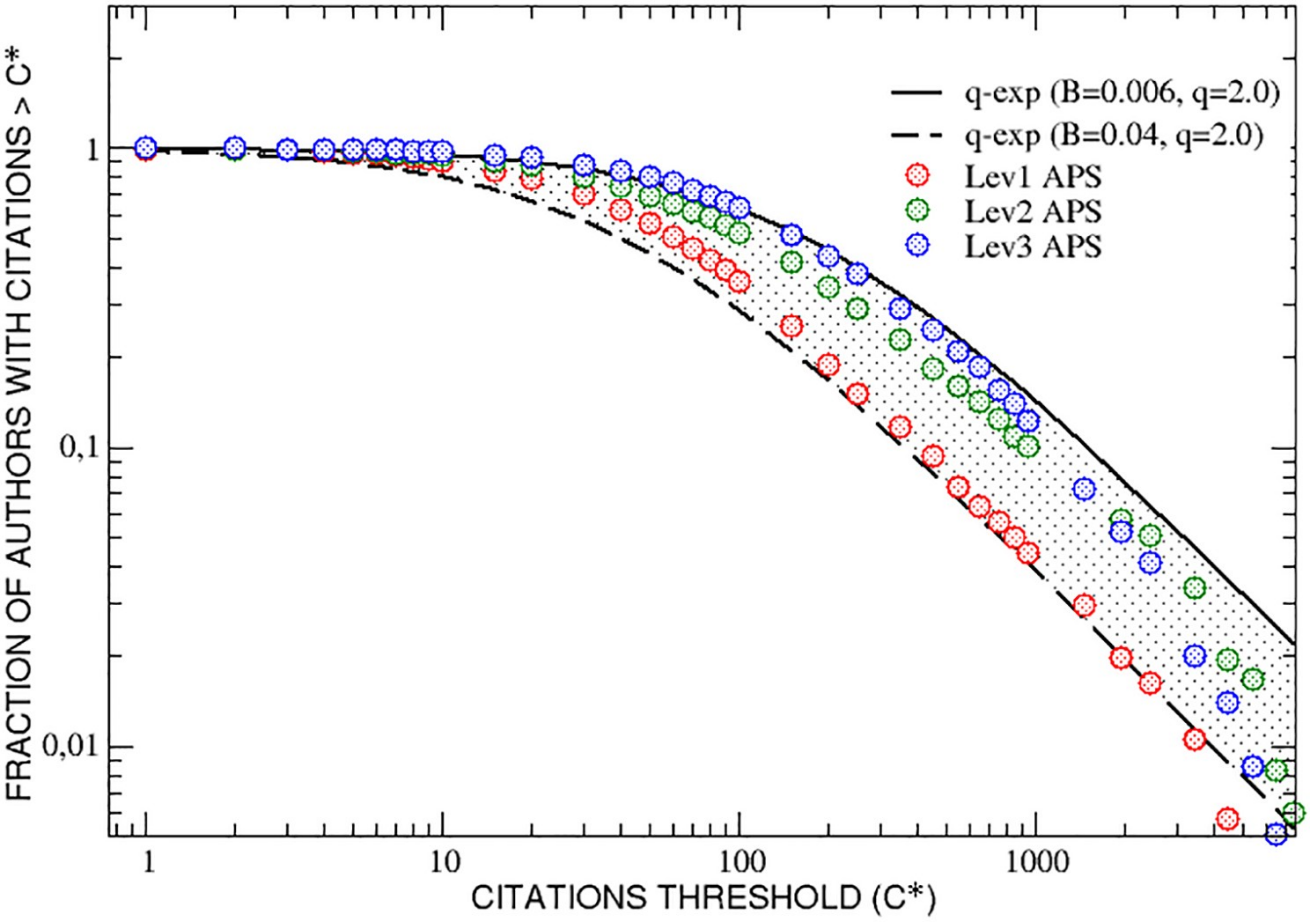
APS data set. The fraction of researchers who have collected, during their careers, a number of citations greater than an increasing threshold C* is reported as function of C*. The three curves, corresponding to different interdisciplinarity levels, lie inside a range limited by two q-exponential functions with the same value for the entropic index *q* and different values of *B* as reported.

In order to better appreciate the differences between the three interdisciplinarity groups from the point of view of their citations scores, it is convenient to take into account, for each group, the fraction of authors who have collected, during their whole careers, a number of citations greater than an increasing threshold C*. In Fig 3 such a cumulative citation distribution is plotted as function of the threshold C*, for the three interdisciplinarity groups. The fraction of highly interdisciplinary researchers (level 3) is generally greater that the fraction of those of level 2 and level 1, in particular for threshold values less than C* = 1000. Above this value, a tendency towards a mixing of the three groups is visible; in any case, authors of level 3 stay always above those of level 1. It is also interesting to notice that the three curves lie inside a range limited by two q-exponential functions $y={\left[1-\left(1-q\right)Bx\right]}^{\frac{1}{1-q}}$ [27] with the same value for the entropic index *q* = 2.0 and different values of *B*, see Fig 3.

## Agent based model: Materials and methods

Next, we turned to the development of an agent-based model able to reproduce, under constraints based on APS real data, the publication-reputation-citation dynamics which generates the observed behavior of our cohort of scholars (see Appendix 2 in the Supporting Information for more details about this section).

**Fig 4:**
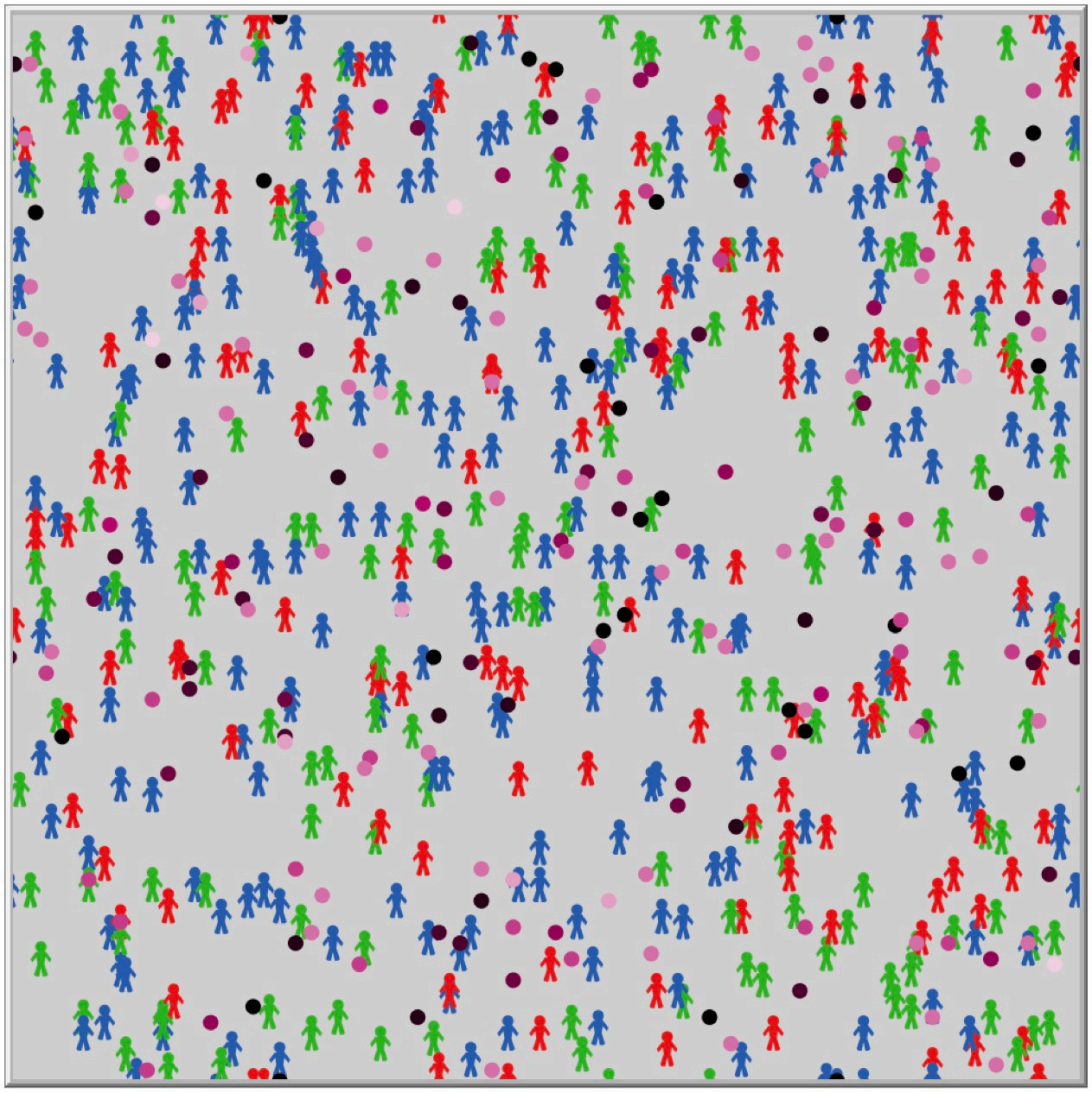
A simplified depiction of the initial state of the agent-based model. For clarity, the figure represents only 500 individuals but the simulations considered the whole cohort of *N* = 7303 researchers active in the period of 30 years taken into account in the analysis of the APS data set. The ‘world’ is a 2D box with periodic boundary conditions.

We start by choosing the initial setup of the model in order to take into account some of the real features of the authors considered in the APS data set. In Fig 4 we show the 2D model world where the *N* = 7303 APS authors (agents depicted as silhouettes) are randomly assigned a position, fixed during the simulations.

Each simulation has a duration *t*_*max*_ of 30 years, with a time step *t* of 1 year. The agents are divided into the three groups $L_{1}^{APS}$ (in red), $L_{2}^{APS}$ (in green) and $L_{3}^{APS}$ (in blue), with sizes *N*_1_, *N*_2_ and *N*_3_ respectively, according with the value of their real interdisciplinary index $I_{i}^{APS}=D_{i}^{APS}\times d_{i}^{APS}$. Each agent is further characterized by the following other variables:

- a fixed talent *T*_*i*_ ∈ [0, 1] (intelligence, skill, endurance, hard-working, …), which is a real number randomly extracted at the beginning of the simulation from a Gaussian distribution with mean *m*_*T*_ = 0.6 and standard deviation *σ*_*T*_ = 0.1;
- an array ${\vec{P}}_{i}\left(t\right)$, whose elements are the papers published by the author *A*_*i*_ at time *t*; the size *P*_*i*_(*t*) of the array ${\vec{P}}_{i}\left(t\right)$ will give the total number of published papers at that time;
- an array ${\vec{C}}_{i}\left(t\right)$, whose elements are the citations received by each paper present in ${\vec{P}}_{i}\left(t\right)$ at time *t* (thus, ${\vec{C}}_{i}\left(t\right)$ and ${\vec{P}}_{i}\left(t\right)$ have the same size); *C*_*i*_(*t*) will be the total number of citations received by all these papers at that time;
- an array ${\vec{R}}_{i}\left(t\right)$ whose 10 elements are the reputation levels reached by the author *A*_*i*_ in each one of the PACS classes; these reputation levels are real numbers, included in the interval [0, 1], which increase as function of the number of papers published in the corresponding disciplinary fields.

The virtual world also contains *N*_*E*_ = 2000 event-points which, unlike the authors, randomly move around during the simulations. They are colored with different shades of magenta, one for each of the 10 PACS classes, and the relative abundance of points belonging to a given class is fixed in agreement with the information of the APS data set (also their total number *N*_*E*_ was calibrated on the real data). Events represent random opportunities, ideas, encounters, intuitions, serendipitous events, etc., which can periodically occur to a given individual along her career, triggering a research line along one or more fields represented by the corresponding PACS class. In this respect, each author owns a “sensitivity circle” representing the spatial extension of their sensitivity to the event-points. The radius of these circles is different for the three interdisciplinarity groups $L_{1}^{APS}$,$L_{2}^{APS}$, $L_{3}^{APS}$ and can be determined through a calibration with real data. Each author, depending on her own group, is sensitive only to the event-points corresponding to the “fertile” PACS which are present in her array ${\vec{D}}_{i}^{APS}$ (we will define these points as “special” points for that author).

The publication-reputation-citation dynamics of this community of authors is quite simple.

- Every year, a check is performed over all the authors in order to verify what type and how many special event-points fall inside their “sensitivity circles”. If, for a given author *A*_*k*_ at year *t*, it results that $0\leq D_{k}\left(t\right)\leq D_{k}^{APS}$ special points are in her circle, the researcher’s talent *T*_*k*_ is compared with a random real number *r* ∈ [0, 1].
- If *r* < *T*_*k*_ (i.e. with probability equal to her talent) the number *P*_*k*_(*t*) of her published papers becomes equal to
  $$\begin{array}{c} P_{k}\left(t\right)=P_{k}\left(t-1\right)+\Delta P_{k} \end{array}$$ (2)
  where Δ*P*_*k*_ is an integer quantity, randomly extracted from a Gaussian distribution with mean $m_{P_{k}}=\mu P_{k}\left(t-1\right)$ and standard deviation $\sigma_{P_{k}}=\gamma P_{k}\left(t-1\right)$. The increment Δ*P*_*k*_ is thus proportional to the number of papers already published by *A*_*k*_ during the previous year *t* − 1 (the coefficients *μ* and *γ*, equal for all the agents, are fixed through a calibration with the APS data).
- All the newly published papers will be added to the array ${\vec{P}}_{k}\left(t\right)$ and each of them will be characterized by the PACS classes corresponding to the event-points that fell in the sensitivity circle at year *t*. Thanks to these new publications, author *A*_*k*_ also increases her reputation in each of the disciplinary fields corresponding to their PACS (i.e. the corresponding elements of the array ${\vec{R}}_{k}\left(t\right)$ will be updated).
- Finally, based on the total number *P*_*k*_(*t*) of published papers at time *t* and on her reputation reached at that time, author *A*_*k*_ yearly updates the elements *c*_*j*_(*t*) (*j* = 1, …, *P*_*k*_(*t*)) of her citations array ${\vec{C}}_{k}\left(t\right)$ with the following rule:
  $$\begin{array}{c} c_{j}\left(t+1\right)=c_{j}\left(t\right)\left(1+{\bar{R}}_{j}\right) \end{array}$$ (3)
  In other words, her *j*-th paper will gain citations, at time *t* + 1, depending on both its previous citation score *c*_*j*_(*t*) and the average reputation ${\bar{R}}_{j}$ of the author in the disciplinary fields corresponding to the PACS present in the paper. Therefore, the overall increase in citations for the author *A*_*k*_ at time *t* + 1 will be
  $$\begin{array}{c} C_{k}\left(t+1\right)=C_{k}\left(t\right)+\sum_{j=1}^{P_{k}\left(t\right)}c_{j}\left(t+1\right) \end{array}$$ (4)

At the end of the simulation, i.e. for *t* = *t*_*max*_, a generic author *A*_*k*_ will have cumulated a certain number *P*_*k*_(*t*_*max*_) of papers and a certain number *C*_*k*_(*t*_*max*_) of citations, depending on her ability in exploiting the opportunities offered by the random occurrence of event-points within her sensitivity circle. Since this ability is parameterized by the talent, the final success of the researchers—in terms of published papers and cumulated citations—will be influenced by both talent and luck (serendipitous events).

## Numerical results

In this section we are interested in verifying if our model is able to capture the stylized facts already observed in the APS data set, with particular regard to the role of interdisciplinarity. Before going on, it is important to note that the agent-based model allows one to average the number of PACS present in each of the *P*_*i*_(*t*_*max*_) publications of a given author *A*_*i*_ at the end of a simulation, enabling the calculation of the dynamical counterpart of the real parameter $d_{i}^{APS}$, i.e. the new parameter $d_{i}^{sim}\left(t_{max}\right)$. By multiplying this parameter for the real $D_{i}^{APS}$, it is possible to update the interdisciplinarity index $I_{i}^{APS}$ of Eq 1—assigned at the beginning of the simulation on the basis of the real APS data—therefore obtaining the new (simulated) index

$$\begin{array}{c} I_{i}^{sim}=D_{i}^{APS}\times d_{i}^{sim} \end{array}$$ (5)

This index, which quantifies the effective interdisciplinarity level reached by each author at the end of a simulation, will allow, in turn, to update also the membership of the authors to the three interdisciplinarity groups, which now become $L_{1}^{sim}$, $L_{2}^{sim}$ or $L_{3}^{sim}$.

**Fig 5:**
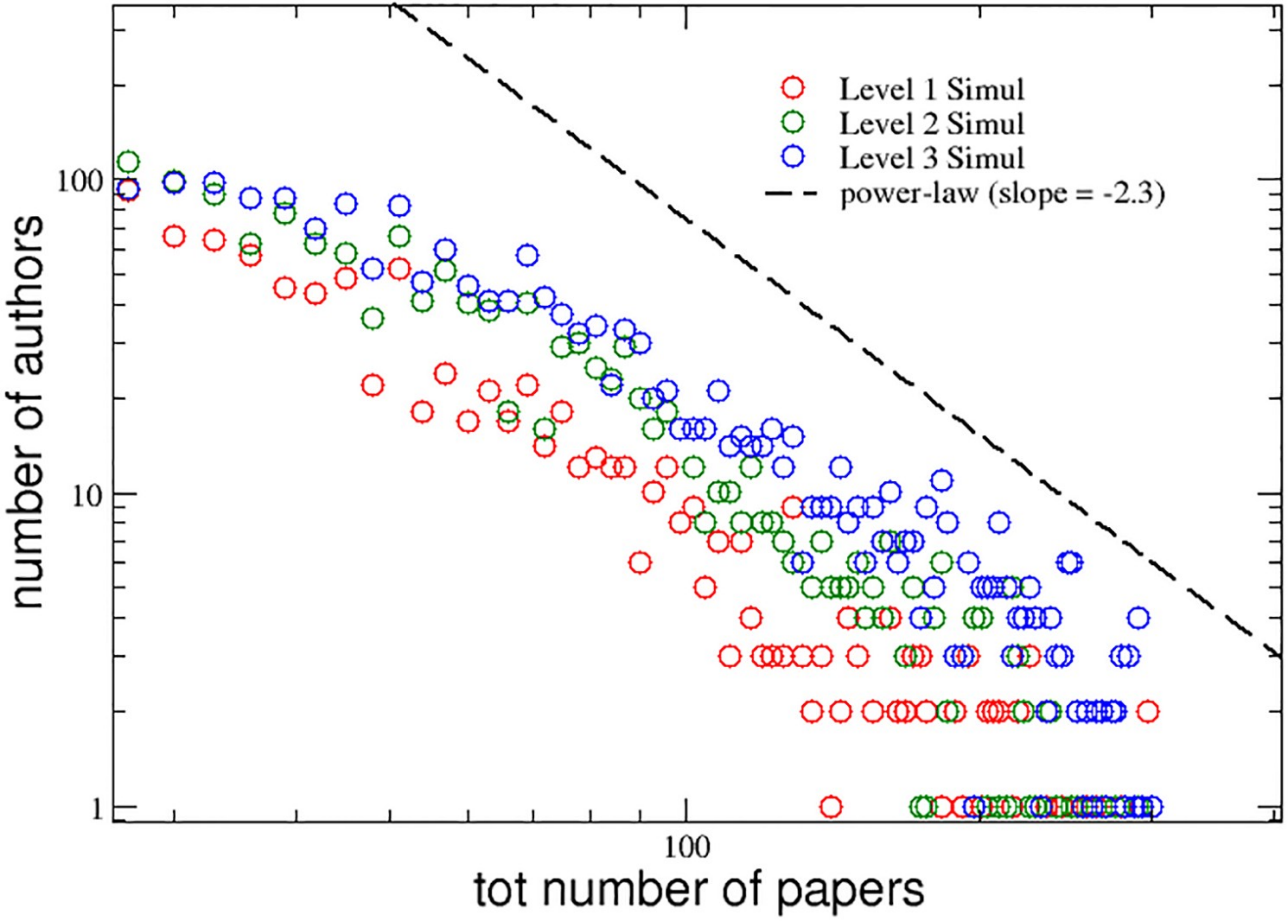
Model simulation. Distributions of the total number of papers published, during their simulated careers, by the authors of the three groups with increasing levels of interdisciplinarity. A power-law curve with slope -2.3 is also reported for comparison (dashed line).

As function of these groups, i.e. of the three corresponding interdisciplinarity levels, we plot in Fig 5 the distributions of the total number *P*_*i*_(*t*_*max*_) of papers published by the *N* authors at the end of a typical simulation. We adopt the same colors as in Fig 1: red, green and blue, respectively. It is evident from the plot, that the proposed model is able to reproduce the same kind of behavior observed for the real APS data set: again, the degree of interdisciplinarity seem to have a strong positive correlation with the productivity of the authors and also the tails of the three distributions follow a power-law trend with the same slope of −2.3 found for the APS data set.

**Fig 6:**
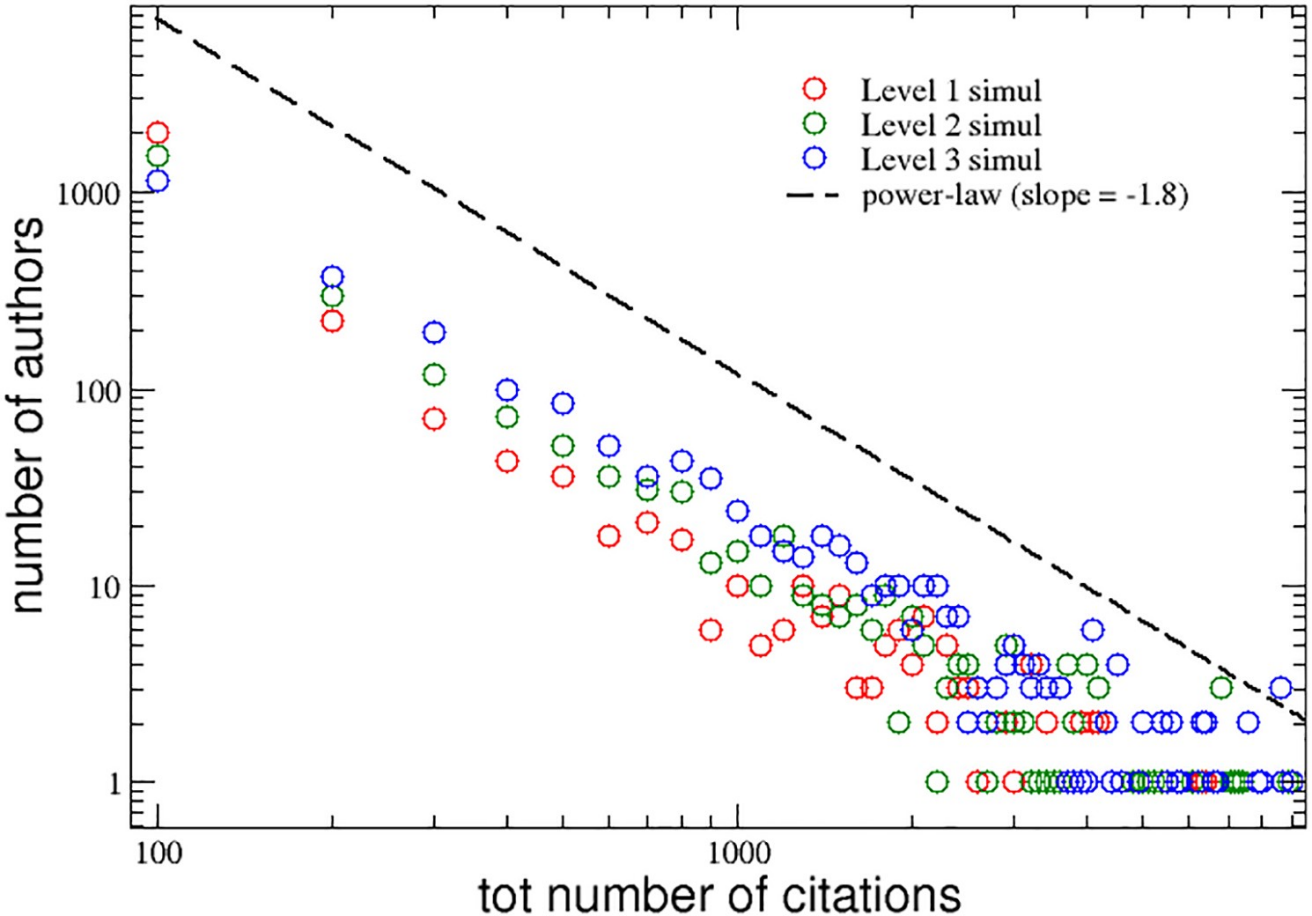
Model simulation. Distributions of the total number of citations cumulated, during their simulated careers, by the authors of the three groups with increasing levels of interdisciplinarity. A power-law curve with slope -1.8 is also reported for comparison (dashed line).

An analogous agreement with the APS data can be observed in Fig 6, where we show the distributions of the total number *C*_*i*_(*t*_*max*_) of citations cumulated by the authors of the three groups during the simulation of their careers. Also in this case, the scientific impact seems strictly correlated with the interdisciplinarity propensity of the researchers. Moreover, the tails of the three distributions follow the same power-law behavior observed for the APS data (see Fig 2), with a slope of −1.8.

**Fig 7:**
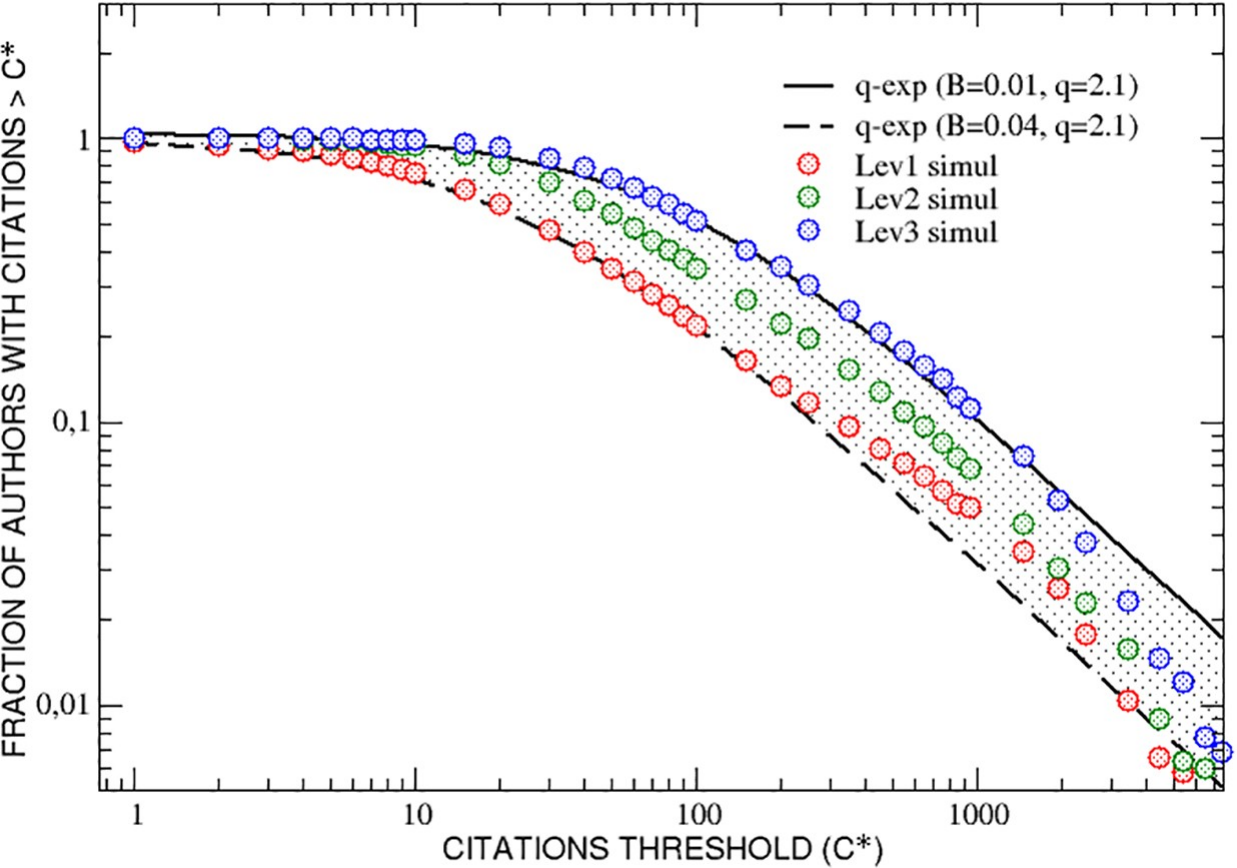
Model simulation. The fraction of researchers who have collected, during their simulated careers, a number of citations greater than an increasing threshold C* is reported as function of C*. The three curves, corresponding to different interdisciplinarity classes, lie inside a range limited by two q-exponential functions with the same value for the entropic index *q* and different values of *B*.

It is also interesting to plot, as in the previous section, the fraction of authors of the three interdisciplinarity groups who have collected, during their whole simulated careers, a number of citations greater than an increasing threshold C*. In Fig 7 it results that the fraction of highly interdisciplinary researchers (level 3) is greater than the fraction of those of level 2 for all values of C*, and the latter is—in turn—always greater than the fraction of level 1 authors. A mixing of the three groups is visible only for very high values of the citation score. As observed in Fig 3, also in this case the three curves fall inside a range limited by two q-exponential functions with a value of the entropic index, *q* = 2.1, very similar to that obtained for the APS data.

**Fig 8:**
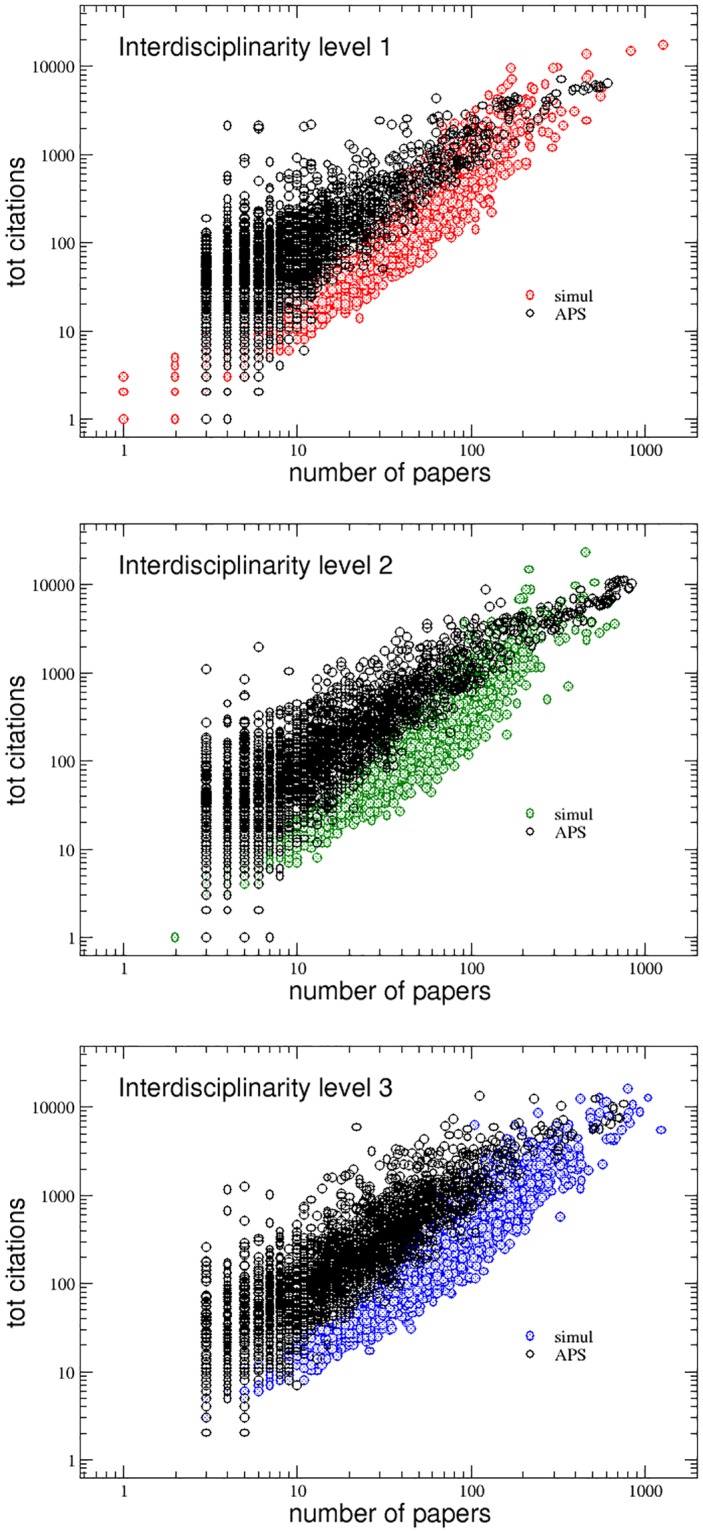
The total number of citations of the researchers of the three interdisciplinarity levels as function of the total number of papers they published in their careers. The figures show that the agent-based model numerical simulations are able to reproduce the positive correlation between these two quantities in all the three groups.

Finally, in the panels of Fig 8, the positive correlation between *P*_*i*_(*t*_*max*_) and *C*_*i*_(*t*_*max*_) for the APS data set and the agent-based model simulation is shown for the three interdisciplinarity levels. The agreement between real and simulated data is remarkable. The only feature, visible in the APS data, which the model does not reproduce, is the presence of authors who published just a few papers (even below 10) but with a very high number of citations. Such an occurrence, characterizing in particular the interdisciplinarity level 1, is probably unpredictable since, as it has been shown in a recent study [17], scientists have the same chance of publishing their biggest hit at any moment in their career and even less productive authors have a chance of publishing very cited papers.

Summarizing, the simulations performed with our agent-based model correctly reproduces the main stylized facts observed in the analysis of the APS data set, confirming that the level of interdisciplinarity plays an important role in determining the scientific success of an author during her academic career. It is important to stress that the calibration of the model with the real data make the output of a single simulation run very robust, despite the differences due to the random initial setup of several model parameters (such as the initial collocation of the authors around the world, their talent distribution and the position/movement of the PACS event-points). This means that these results can be considered quite general and well representative of the model’s behavior (we checked that they do not change even performing ensemble averages over many runs).

Having established the agreement between experimental and modeled data, we turn to the analysis of variables which are impossible to observe directly from the real data. For example, one could wonder if the most successful authors in the three interdisciplinarity groups are also the most talented ones. In a recent numerical study about the causes behind the achievement of success in our life [19], it has been shown that individual talent is necessary but not sufficient to become rich or to climb the social ladder: luck plays a fundamental role and very often moderately gifted, but very lucky, people surpass highly talented, yet unlucky, individuals.

**Fig 9:**
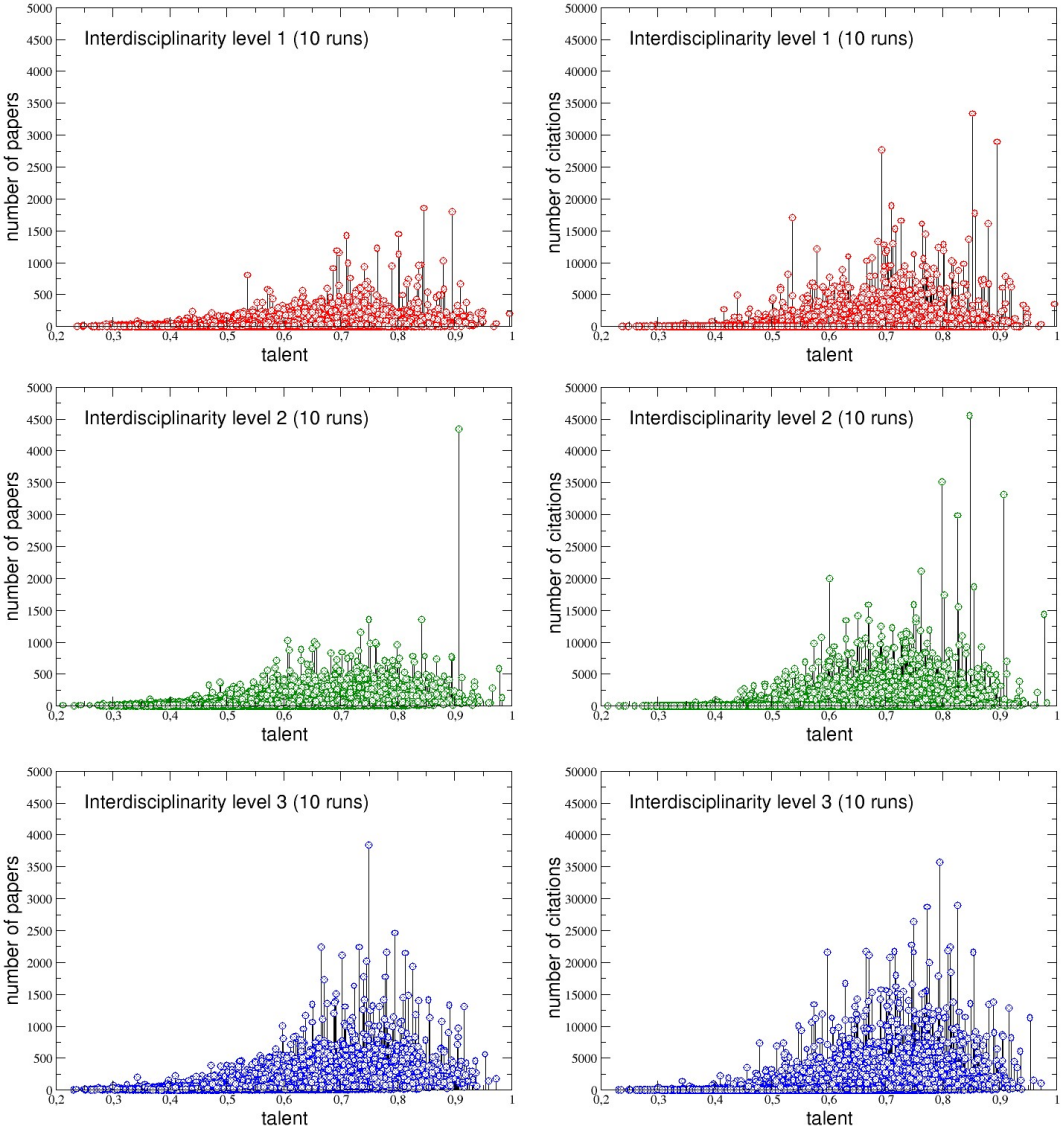
Papers and citations vs talent, collected over 10 replica runs of the same numerical simulation. Each circle in the figures represents the total number of papers (left column) or the total number of citations (right column) cumulated by each author of the three interdisciplinarity groups in each of the 10 runs, reported as function of the corresponding talent. These plots indicate in a clear way that the most successful individuals are never the most talented ones.

Finally, we show that this counterintuitive feature holds also in the scientific context addressed here. We performed 10 replica runs of our agent-based model, with the same calibration based on real APS data, but with different distributions of the talent among the 7303 authors and with different initial positions for both the agents and the 2000 event-points. In Fig 9 we plot the final number of papers (left column) and the final number of citations (right column) cumulated by each author belonging to the three interdisciplinarity groups during all the 10 simulations, as a function of their talent. The results indicate that very talented people—for example researchers with a talent *T*_*i*_ > 0.9—are very rarely the most successful ones, regardless the interdisciplinarity group they belong. Rather, their papers or citations score stays often quite low. On the other hand, scientists with a talent just above the mean—for example in the range 0.6 < *T*_*i*_ < 0.8—usually cumulate a considerable number of papers and citations. In other words, the most successful authors are almost always scientists with a medium-high level of talent, rather than the most talented ones. This happens because (i) talent needs lucky opportunities (chances, random meetings, serendipity) to exploits its potentialities, and (ii) very talented scientists are much less numerous than moderately talented ones (being the talent normally distributed in the population). Therefore, it is much easier to find a moderately gifted *and* lucky researcher than a very talented *and* lucky one.

It is also interesting to note that this effect is more pronounced for authors with a low interdisciplinarity level and progressively decreases by increasing the degree of interdisciplinarity. In order to quantitatively address this last point, let us define as moderately gifted (*N*_*M*_) authors *A*_*i*_ with a talent around the mean, i.e. with 0.5 < *T*_*i*_ < 0.7, and highly talented (*N*_*T*_) those with *T*_*i*_ > 0.8 (i.e. greater than two standard deviations with respect to the mean). Let us also call ${\bar{P}}^{sim}$ and ${\bar{C}}^{sim}$ the average values of, respectively, the final number of papers *P*_*i*_(*t*_*max*_) and the final number of citations *C*_*i*_(*t*_*max*_) cumulated by each author inside the three groups $L_{1}^{sim}$, $L_{2}^{sim}$, $L_{3}^{sim}$. Looking to the details in Table 2, it is evident that, inside each of the three groups, these averages (columns 1 and 5) do increase with the level, highlighting a positive correlation between scientific success and interdisciplinarity analogous to that one already observed for the same quantities calculated for the APS data set and reported in Table 1 (columns 3 and 5). On the other hand, the percentages of highly talented scientists with a final number of papers $P_{i}\left(t_{max}\right)>{\bar{P}}^{sim}$ or with a final number of citations $C_{i}\left(t_{max}\right)>{\bar{C}}^{sim}$, with respect to the same percentages for the moderately gifted one, also increase by increasing the interdisciplinarity level. This is seen by the ratios between the two percentages, *r*_*P*_ = (*N*_*T*_/*N*_*M*_)_*P*_ and *r*_*C*_ = (*N*_*T*_/*N*_*M*_)_*C*_, which increase respectively from 0.06 to 0.1 and from 0.08 to 0.12 going from $L_{1}^{sim}$ to $L_{3}^{sim}$.

**Table 2:** Details about the percentage of moderately gifted (*N*_*M*_) and highly talented (*N*_*T*_) authors whose publications or citations overcome the respective averages, for each of the three groups with increasing interdisciplinarity level.

	${\bar{P}}^{sim}$	*N*_*M*_	*N*_*T*_	*r*_*P*_	${\bar{C}}^{sim}$	*N*_*M*_	*N*_*T*_	*r*_*C*_
$L_{1}^{sim}$	30	18.6%	1.1%	0.06	191	8.7%	0.7%	0.08
$L_{2}^{sim}$	49	18.5%	1.5%	0.08	297	10.3%	1.16%	0.11
$L_{3}^{sim}$	82	18.8%	1.8%	0.10	505	12.1%	1.5%	0.12

## Conclusions

In conclusion, in this paper we have shown, through both a statistical analysis performed on the APS data set and a comparison with the numerical results obtained by an agent-based model (calibrated on the real data), that the attitude to broaden the scope of their researches, mixing different fields of physics, is able to provide more rewards to the scientists, since their productivity and their scientific impact increase with their level of interdisciplinarity. Moreover, averaging over several runs with different initial distributions of talent among all the authors, we have also shown that, very often, moderately gifted researchers reach higher level of scientific success than very talented ones, simply because they have had more opportunities or just because they were luckier. However, the interdisciplinarity level seems to slightly dampen this effect since its increase does enhance the probability of success of highly talented individuals with respect to the moderately talented ones. Due to the generality of the APS data set, we expect that our findings remain valid beyond the considered case study and beyond physics itself. Further applications of the statistical and computational approach introduced in this study to other scientific fields could be also possible. For example, it could be in principle be readily transferred to disciplines adopting tree-based classification schemes, such as MeSH (medicine), MSC (mathematics) or JEL (economics). A more general approach would imply substituting PACS numbers with keywords or with other significant strings appearing either in the title or in the abstract of the papers present in the considered data set. We are actually working along these directions and other works are in preparation.

## Supporting information

S1 File APPENDIX 1: APS data set analysis. (PDF)

S2 File APPENDIX 2: The agent-based model. (PDF)
