# Supplementary material for: Exploring the role of interdisciplinarity in physics: Success, talent and luck
Source: PLoS One. 2019 Jun 26;14(6):e0218793. doi: 10.1371/journal.pone.0218793 (PMC6594681; doi:10.1371/journal.pone.0218793)
Supplement: S1 File — (PDF) [file pone.0218793.s001.pdf]

APPENDIX 1: APS DATA SET ANALYSIS

We give in this Appendix 1 additional details about the methodologies behind the mining of the American Physical Society (APS) data set from which we have got the results described in the Main Paper (MP).

1.1 APS Data Set Structure

The APS data set consists of all the publications of American Physical Society from 1893 to 2013. Each publication is represented through a JSON file storing information about authors, their affiliations, the journal and the PACS or keywords associated to the paper. The database has of more than 550000 publications. A critical aspect relative to the APS data set relies on its noise due to the lexical heterogeneity. Lexical heterogeneity occurs when the tuples have identically structured fields across databases, but the data use different representations to refer to the same real-world object. In our case, authors and affiliations are stored using different conventions in each JSON file. Therefore, the same author, or affiliation can be represented in a different format (i.e. Mark John Smith or Mark J. Smith or Smith M.). Based on this consideration, two records can be considered *equivalent* if they are semantically equal. The similarity between records is computed by metrics which measure the semantic equivalence through a score. Record pairs with high similarity scores (above a specified threshold) are treated as duplicates.

In addition to the accuracy of classifying records pairs into matches and mismatches, the central issue consists of improving the speed of comparisons. Indeed, cleaning such data before its usage is a mandatory step to avoid redundant and noisy information and affect the reliability of further analysis. To remove duplicate entries we decided to compare two strings (i.e. affiliations of authors) using q-grams [1] in connection to Jaccard Similiarity [2]. The Jaccard Similarity of two sets a and b is defined as $sim(a,b) = \frac{|a \cap b|}{|a \cup b|}$ ranging from 0 to 1. Practically, we extracted from each string q-grams of length 2 ($q = 2$, for both authors and affiliations), then we claim two authors to be the same when their Jaccard Similarity is greater than a threshold set equal to 0.6. Similarly two affiliations have been declared to be the same if their similarity is greater than the threshold 0.66. These two threshold have been empirically established on a sample of data from APS data set by minimizing the ratio of false negatives (same author/affiliation but we consider the two authors/affiliations as different) and false positives (different authors/affiliations but considered the same author).

Due to the large number of authors and affiliation we experienced a computational bottleneck due to the quadratic time needed to perform all possible pairwise comparisons. To make such a cleaning step feasible we implemented the similarity computation in connection to the Locality Sensitive Hashing (LSH) [3]. LSH is an algorithmic methodology which makes use of hashing, that is able to fast identify similar pairs of objects without comparing them directly. Using such a technique we were able to reduce the computational effort from quadratic to linear. All the code have been developed in Php and the data, once cleaned, were stored into the relational database MySQL (v. 5.1). Further manipulation and analysis of cleaned data were done using R language.

The measure of the level of interdisciplinarity of the authors (in the discussion we will refer to them also as 'researchers') is based on the APS's PACS ('Physics and Astronomy Classification Scheme'). This scheme consists of a hierarchic partition of the publications in research areas of physics. Any PACS code has four hierarchic levels of increasing specificity: a first and a second digit composing a two-digit number, another two-digit number and a string of characters (e.g. 14.70.Bh). In particular, we work with the less specific hierarchic level, made up by the ten areas of research each corresponding to one of the ten different first digits (0, 1, ... 9; or equivalently 00, 10, ... 90) of the first two-digit number in the PACS code:

00 - GP : General Physics
10 - EPF : Physics of Elementary Particles and Fields
20 - NP : Nuclear Physics
30 - AMP : Atomic and Molecular Physics
40 - EOAHCF : Electromagnetism, Optics, Acoustics, Heat Transfer, Classical Mechanics, and Fluid Dynamics
50 - GPE : Physics of Gases, Plasmas, and Electric Discharges
60 - CM:SMT : Condensed Matter: Structural, Mechanical and Thermal Properties
70 - CM:EEMO : Condensed Matter: Electronic Structure, Electrical, Magnetic, and Optical Properties
80 - IPR : Interdisciplinary Physics and Related Areas of Science and Technology
90 - GAA : Geophysics, Astronomy, and Astrophysics

Since the APS database regards only the physics' domain, this choice is led by our purpose of identifying an actual interdisciplinarity attitude in the researchers' production. Any published paper can have one or more PACS codes

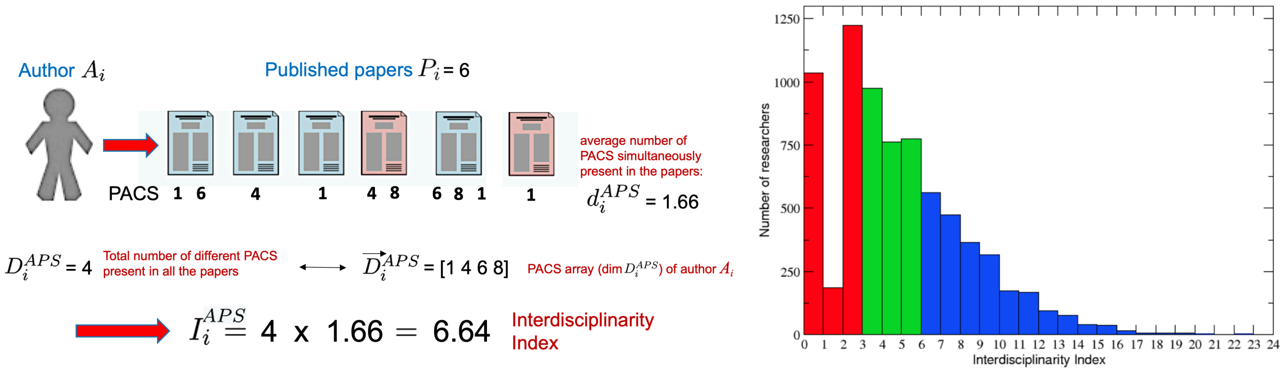

FIG. S1: (Left Panel) An example of calculation of the interdisciplinarity index for an imaginary author A_i who published 6 papers. (Right Panel) Histogram of the interdisciplinarity index I^{APS} for the $N = 7303$ researchers interested by our study. The three different interdisciplinarity levels are represented with different colors: red (level 1), green (level 2) and blue (level 3). The bar for I^{APS} between j and $j+1$ represents the number of researchers with $I^{APS} \in]j, j+1]$. In particular, the first two bars contains only researchers with $I^{APS} = 1$ and $I^{APS} = 2$, respectively.

assigned to it and according to our choice we assign different PACS codes to a paper only if these codes differ on the first digit; otherwise, we pile them up on a single code. In this way we assign to each paper a number of PACS codes that is equal to the number of the different broad - less specific - areas related to it. From what has been said, is understood that only PACS classified papers are considered.

1.2 Researchers Classification

Having at our disposal the PACS coded areas of all the papers, we may use them to define an index that helps us to quantify the variety of disciplines (areas) interested by the scientific production of any researcher. This variety is two-fold: a researcher may explore many different areas one by one, i.e. producing on many different PACS codes through papers with assigned only one code at a time; or she may explore few different areas but jointly, i.e. producing papers having more codes assigned together. In other words, a researcher's production can be interdisciplinary either because of the total number of areas that it interested, or because of the average number of areas jointly interested in one of its typical paper. As it is going to be evident, apart from an obvious constraint, these two degrees of interdisciplinarity are independent of each other. This observation led us to define an interdisciplinary index I_k^{APS} for the researcher A_k as

$$I_k^{APS} = D_k^{APS} \times d_k^{APS}$$

where d_k^{APS} is the average number of different PACS codes jointly present in each paper of the considered author and $D_k^{APS} \in [1, 10]$ is the total number of different PACS codes present in all the papers of the same author. One can also imagine to assign to A_k an array $\vec{D}_k^{APS}$ containing all the D_k^{APS} PACS numbers present in her papers. The constraint mentioned above is the mere condition $d_k^{APS} \leq D_k^{APS}$ for any k. In fact, the maximum number of PACS codes assignable to a paper is five, so, at least in principle, the maximum value of I^{APS} is 50, with $d^{APS} = 5$ and $D^{APS} = 10$. In practice, for our data set, the maximum value found for I is 23, with $d^{APS} = 3.286$ and $D^{APS} = 7$. In the left panel of Fig.S1 an example of calculation of the interdisciplinarity index for a hypothetic author A_i is presented. This author has published $P_i = 6$ papers, each one with different PACS numbers (1-6, 4, 1, 4-8, 6-8-1, 1, respectively). The corresponding PACS array is thus $\vec{D}_i^{APS} = [1468]$, $D_i^{APS} = 4$ and $d_i^{APS} = 1.66$. Therefore, her interdisciplinarity index will be $I_i^{APS} = 6.64$.

Once the interdisciplinarity index has been calculated for each researcher, we have distributed all the 7303 authors - resulted from the filtering procedure explained below - into three groups of different interdisciplinarity level (see right panel of Fig.S1):

- Level 1 (L_1^{APS}): $1 \leq I_k^{APS} \leq 3$ ($N_1 = 2445$ researchers of low interdisciplinarity level)
- Level 2 (L_2^{APS}): $3 < I_k^{APS} \leq 6$ ($N_2 = 2511$ researchers of medium interdisciplinarity level)

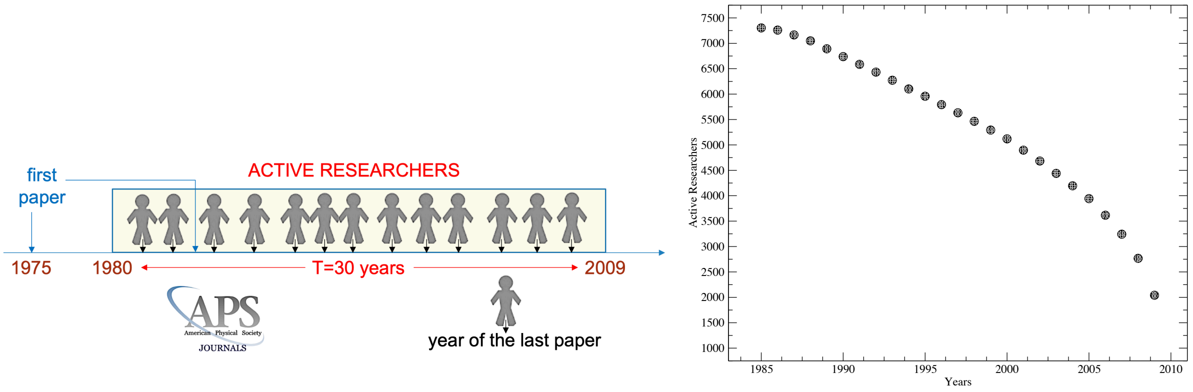

FIG. S2: (Left Panel) The active researchers considered in the APS data set analysis, see text. (Right Panel) Time evolution, year by year, of the number of still active researchers. A linear decrease is found from 1987 to 2002, with 165 leaving researchers a year, on average. After 2002 a kind of cut off acts, maybe due to the their ages. The 28% of them is still active at the end of the thirty years.

- Level 3 (L_3^{APS}): $I_k^{APS} > 6$ $\;(N_3 = 2347$ researchers of high interdisciplinarity level)

The separation values between the levels have been chosen to have the three groups with comparable sizes and, for the set of researchers used here, the best values came out to be 3 and 6, if we want them as easy-to-remind integer numbers. To note that for the level 1, because of the condition $d_k^{APS} \leq D_k^{APS}$, the index I_k^{APS} cannot take value in the open interval (1,2).

The 7303 researchers on which we have conducted our analysis are the remaining ones of a filtering procedure conceived to study appropriately the researchers' careers over a period of thirty years, from 01/01/1980 to 31/12/2009. The first requirement of the filtering is that a researcher must have produced her first paper in the period ranging from 01/01/1975 to 31/12/1985 (see the left panel of Fig.S2). This ensures that all the researchers in the set started their careers in a quite short period, so avoiding that the possible premature end of the production activity of a researcher is due to her age. In this way, unless one started to produce in old age, that is a pretty remote possibility, all the researchers in the set have comparable ages. Moreover, the PACS classification was implemented from 1975 onwards, enabling us to refer only to papers published starting from that year. The second requirement is that a researcher must have produced a minimum number of (PACS classified) papers, that we chose to be 3. The third, last, requirement is related to the way in which the raw APS database at our disposal has been cleaned (extensively explained in the specific section).

Briefly, at each author's name has been given an author identification code and the same code has been assigned to different names if they were similar enough. We refer to the authors' name associated with the same author code as aliases of that author. We ruled out those author codes with more than one alias associated to it. We realized, indeed, that not enough rarely happened that two aliases referred to two actually different authors (with similar names, unfortunately), leading us to overestimate the productivity and the impact of the unique author code which they were assigned to. These three requirements filtered the database leaving us with 7303 initial author codes, corresponding to the 7303 actually different researchers on which we have performed our analysis.

Looking at the last published paper by each researcher, apart of a late cut off, an approximately linear decrease in time of the number of active researchers came out. Starting with all the 7303 researchers active in 1985, we end up with 2041 of them still active in 2009 (Fig.S2, right panel).

1.3 Scientific Impact Analysis

The scientific production in the period 1980-2009 of the 7303 selected researchers consists of 89949 (PACS classified) papers. These are distributed in a slightly different way over the three defined classes of interdisciplinarity, see the left panel of Fig.S3. In all of them one can note long tails of a few dozen of researchers with an exceptional productivity, but in general interdisciplinarity seem to have a positive influence on the average productivity of a scientist. Some examples of the increase in the scientific production during single excellent careers for the three classes is shown in

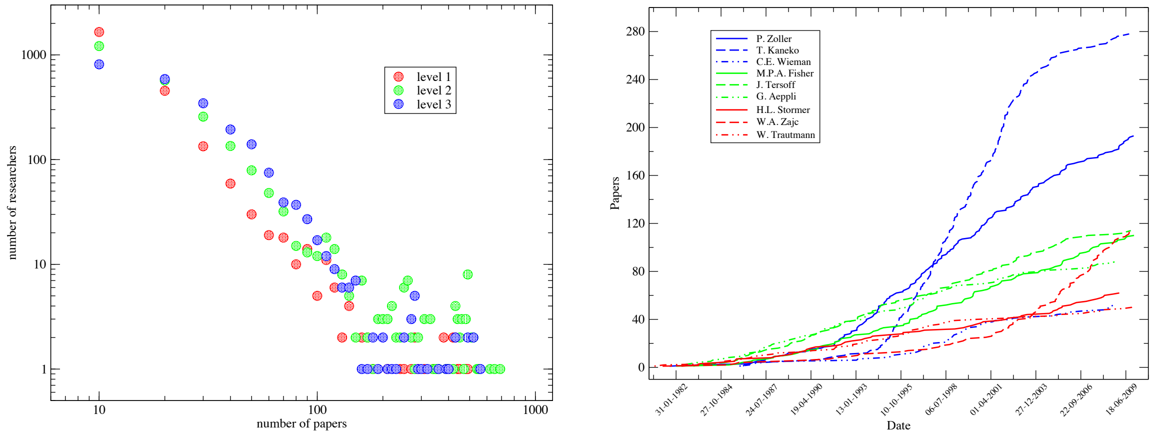

FIG. S3: (Left Panel) Papers distribution for the three defined classes of interdisciplinarity, each represented with a different color: red (level 1), green (level 2) and blue (level 3). A tail of scarse statistics starts for numbers of researchers with more than about 150 published papers. (Right Panel) Examples of scientific production in some excellent careers for the three interdisciplinarity classes.

	authors	papers	PpA	avg. PpA (st. dev.)
level 1	2445	18832	7.70	15.38 (37.22)
level 2	2511	35892	14.29	29.35 (67.18)
level 3	2347	50947	21.71	27.30 (42.26)

TABLE S1: Statistical indicators of the 89949 published papers over the three defined classes of interdisciplinarity. A paper is counted in more than one class if it is coauthored by researchers belonging to different classes, so the sum of the reported numbers of papers exceeds 89949. A positive correlation between scientific production and interdisciplinarity level is found: the number of papers per researcher (PpA = papers/authors) increases quite strongly as the interdisciplinarity level grows.

the right panel of Fig.S3, where the cumulated number of papers is reported as function of time.

		PACS Area									
		00	10	20	30	40	50	60	70	80	90
Level 1	papers	609 (3.23%)	4892 (25.98%)	5989 (31.80%)	1488 (7.90%)	305 (1.62%)	720 (3.82%)	1518 (8.06%)	5232 (27.78%)	93 (0.49%)	236 (1.25%)
	researchers	231 (9.45%)	782 (31.98%)	811 (33.17%)	277 (11.33%)	128 (5.24%)	197 (8.06%)	475 (19.43%)	744 (30.43%)	68 (2.78%)	98 (4.01%)
Level 2	papers	3244 (9.04%)	7466 (20.80%)	6703 (18.68%)	3006 (8.38%)	1715 (4.78%)	1064 (2.96%)	6101 (17.00%)	15013 (41.83%)	1361 (3.79%)	1121 (3.12%)
	researchers	1032 (41.10%)	849 (33.81%)	794 (31.62%)	685 (27.28%)	528 (21.03%)	220 (8.76%)	1213 (48.31%)	1317 (52.45%)	696 (27.72%)	367 (14.62%)
Level 3	papers	12397 (24.33%)	6056 (11.89%)	4700 (9.23%)	6430 (12.62%)	7265 (14.26%)	1813 (3.56%)	14159 (27.79%)	21461 (42.12%)	5612 (11.02%)	1867 (3.66%)
	researchers	1705 (72.65%)	743 (31.66%)	699 (29.78%)	1216 (51.81%)	1348 (57.44%)	503 (21.43%)	1790 (76.27%)	1790 (76.27%)	1447 (61.65%)	460 (19.60%)

TABLE S2: Distribution of the researchers of each interdisciplinarity level and their papers through the ten PACS coded areas.

A confirm of the positive correlation between scientific production and interdisciplinarity level is shown in Table S1. Comparing the number of papers per author and the (real) average number of papers per author (avg. PpA), we also find a stronger presence of coauthoring in the level 1 and level 2 classes than in the level 3 class. This is due mainly to

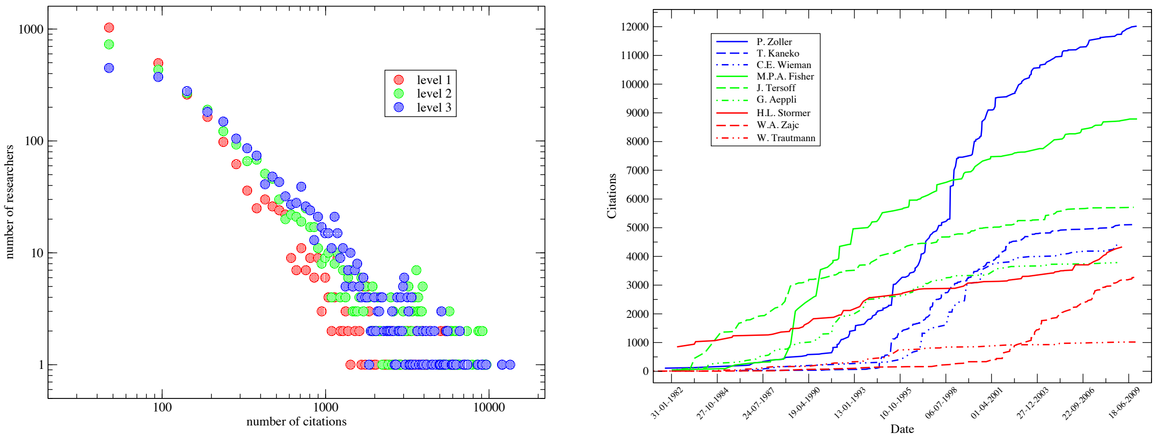

FIG. S4: (Left Panel) Citations distribution for the three defined classes of interdisciplinarity, each represented with a different color: red (level 1), green (level 2) and blue (level 3). (Right Panel) The same careers shown in Fig.S3 are here addressed in terms of time evolution of scientific impact.

	authors	papers	citations	CpA	avg. CpA (st. dev.)
level 1	2445	18832	230448	94.25	217.52 (598.48)
level 2	2511	35892	515635	205.35	458.44 (1121.24)
level 3	2347	50947	843292	359.31	479.07 (997.75)

TABLE S3: Statistical indicators of the citations received by the authors and their papers for each of the three defined classes of interdisciplinarity. All these citations divide slightly differently for each class (Fig. S4, left panel). A positive correlation between scientific impact, in terms of citations received, and interdisciplinarity level is found: the number of citations per author (CpA = citations/authors) raises as the interdisciplinarity level increases.

the fact that a lower percentage of researchers of the level 3 class participated to large scientific collaboration, respect to the other two classes.

By looking minutely at their production one finds out that all of them did research in the areas of particle and nuclear physics. More precisely, these researchers took part in large scientific experiments (e.g. BABAR, CLEO, CDF collaborations) during the 2000s. These large collaborations of hundreds of scientists ensure to the participants high rates of scientific productivity of even 60/70 published papers a year, an unachievable goal for the small research groups working in other areas. As proved by the composition of the three interdisciplinarity classes in terms of the ten PACS coded areas - see Table S2 - most of the researchers in our set who are involved in these large collaborations belong to the level 2 class, justifying the heavier tail found for this class compared to those found for the other two classes (Fig.S3).

One easily notes that these indicators clearly underestimate the real productivity of the researchers, but it must be kept in mind that they refer only to (PACS coded) publications on APS and that the actual number of researchers decreased over the thirty years, as shown in Fig.S2.

The 89949 (PACS classified) published papers of the set received a total of 1329374 citations within the APS system in the period 1980-2009. From the point of view of the 7303 researchers, considered as independent, they received a total of 2807368 citations in the same period. All these citations divide similarly among the researchers of each of the three interdisciplinarity classes, as shown in the left panel of Fig.S4. Also in this case, as previously shown for the papers production, we found a positive correlation between scientific impact, in terms of citations received, and interdisciplinarity level (Table S3). Finally, in the right panel of Fig.S4, the increase in the number of citations cumulated by the same excellent careers considered in Fig.S3 is reported as function of time. Notice that not necessarily the best score in terms of published papers does imply the best score in terms of scientific impact and vice-versa.

As a final curiosity, apart from these excellences, let us see some other authors names belonging to the three interdisciplinarity groups of our data set. In particular, in the L_1^{APS} group one find mainly scientist who have been working in nuclear physics, like W. Alberico, U. Lynen, Y.T. Oganessian, W. Trautmann. On the other hand, in the

L_2^{APS} group one can find scientists who worked in various fields, from chaos theory to gravitational waves, or from quantum information to cosmology, as for example C. Grebogi, D. Deutsch, K. Wilson, J.E. Jaffe, L. Smolin, P.C.W. Davies, G. Pizzella. Finally, in the most interdisciplinar L_3^{APS} group, one finds mainly statistical or condensed matter physicists, scientists involved in complex networks and dynamical systems, and also cosmologists or experts of string theory with broad views (P. Bak, A. Coniglio, K. Kaneko, M. Mezard, S. Havlin, D. Sornette, G. Parisi, J. Barrow and B. Greene).

[1] L. Gravano, P. G. Ipeirotis, H. V. Jagadish, N. Koudas, S. Muthukrishnan, and D. Srivastava, in Proceedings of the 27th International Conference on Very Large Data Bases (Morgan Kaufmann Publishers Inc., San Francisco, CA, USA, 2001), VLDB '01, pp. 491-500, ISBN 1-55860-804-4, URL http://dl.acm.org/citation.cfm?id=645927.672200.
[2] P.-N. Tan, M. Steinbach, and V. Kumar, Introduction to data mining (2013).
[3] E. Cohen, M. Datar, S. Fujiwara, A. Gionis, P. Indyk, R. Motwani, J. D. Ullman, and C. Yang, IEEE Transactions on Knowledge and Data Engineering 13, 64 (2001).
