# Supplementary material for: Exploring the role of interdisciplinarity in physics: Success, talent and luck
Source: PLoS One. 2019 Jun 26;14(6):e0218793. doi: 10.1371/journal.pone.0218793 (PMC6594681; doi:10.1371/journal.pone.0218793)
Supplement: S2 File — (PDF) [file pone.0218793.s002.pdf]

APPENDIX 2: THE AGENT-BASED MODEL

Let us address, in this Appendix 2, some details about the agent-based model with which we were able to successful replicate the stylized facts of the APS data set. The model was realized within NetLogo, a very powerful multi-agent programmable environment particularly suitable for the the simulation of the dynamical behavior of complex systems [1].

2.1 Initial setup of the model

In Fig.S1 we show the NetLogo "world" as it appears at the beginning of a generic simulation. It is a squared metric space, with a size of 201×201 patches, where the various agents live and move. Randomly distributed around the world are visible the two main categories of agents of our model: N researchers, with a person-like shape, and N_E PACS event-points, with a point-like shape. Both these agent's types are active elements of the environment, able of interact one among each other.

In the figure we represent only $N = 500$ individuals for a better visualization, but in all the simulations we consider all the $N = 7303$ active researchers, as in the APS data set. These researchers do no move during a simulation and are divided into the three groups L_1^{APS}, L_2^{APS} and L_3^{APS} according with the real values of their interdisciplinary index $I_i^{APS} = D_i^{APS} \times d_i^{APS}$. Therefore, we will find N_1 individuals in the group L_1^{APS} (in red), N_2 in the group L_2^{APS} (in green) and N_3 in the group L_3^{APS} (in blue). During a single simulation run, we will let these researchers to publish papers and collect citations with a periodicity of $t = 1$ year and for a total time interval of $t_{max} = 30$ years, in analogy with the real time period addressed in the APS data set. A first evident approximation of the model is the fact that we will keep the total number of active authors constant during the 30 years, while we know that their number do decrease, as shown in Fig.S2. This will imply an overestimation of the total number of published papers of several authors, but - as we have already stated - we are interested to capture the main stylized facts of the APS data set not the single details (which, of course, would be impossible to reproduce).

Each simulated author A_i is characterized not only by the variables I_i^{APS}, D_i^{APS}, $\vec{D}_i^{APS}$ and d_i^{APS} ($i = 1, ..., N$), which are read from the APS data set, but also by other individual parameters shown in the left panel of Fig.S2 and described in the MP. In particular, to each researcher is assigned a fixed talent $T_i \in [0, 1]$ (intelligence, skill, ...) randomly extracted at the beginning of each simulation run from a truncated Normal distribution with a mean

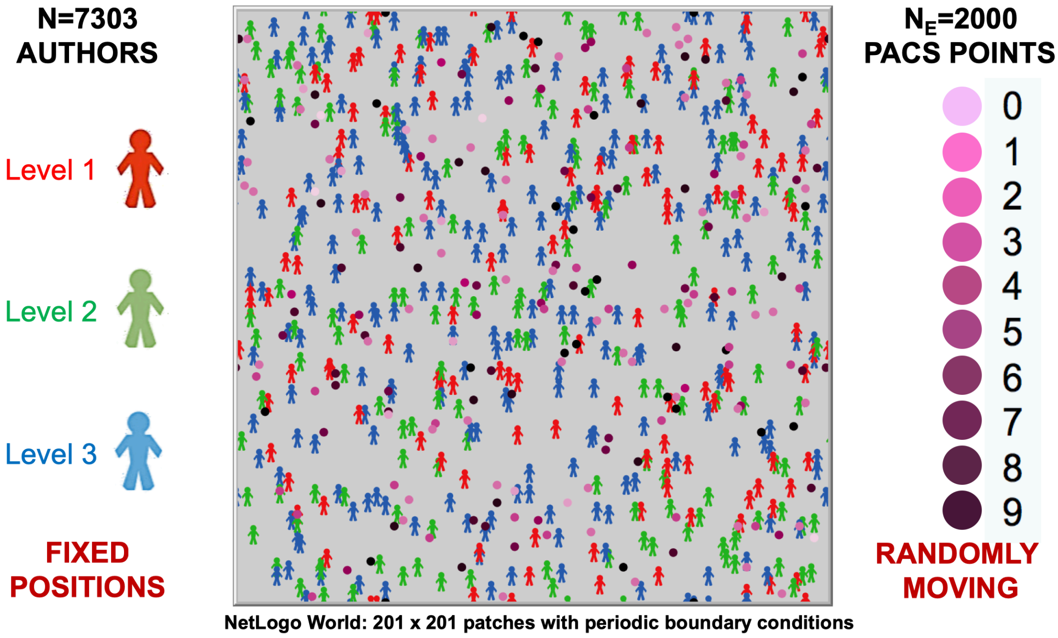

FIG. S1: An example of initial setup for our simulations.

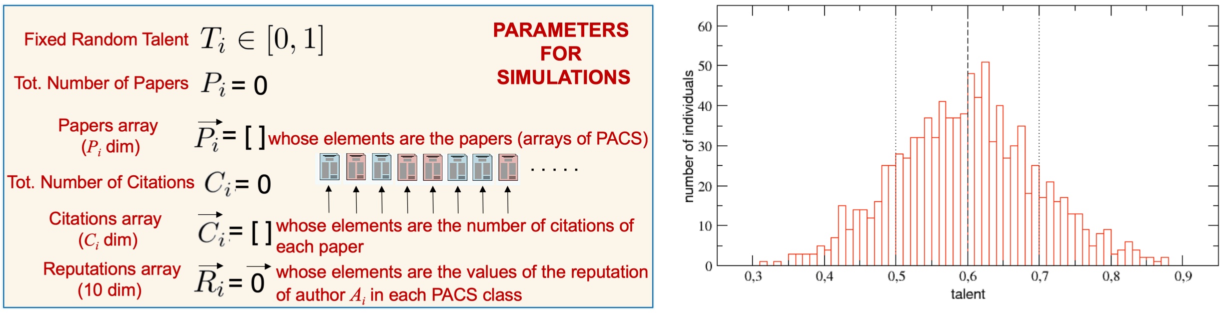

FIG. S2: (Left Panel) Individual parameters which characterize each single simulated author A_i. (Right Panel) Normal distribution of talent among the agents, with mean $m_T = 0.6$ (indicated by a dashed vertical line) and standard deviation $\sigma_T = 0.1$ (the values $m_T \pm \sigma_T$ are indicated by two dotted vertical lines). This distribution does not change during a single simulation run.

$m_T = 0.6$ and a standard deviation $\sigma_T = 0.1$ (see the right panel of Fig.S2). All the other individual parameters start from a null value at $t = 0$ and increase in time during the simulation following opportune dynamical rules.

As we will show in the next subsection, other global parameters need to be introduced in the model and calibrated through the comparison with the real APS data.

2.2 Calibration of the model

The first global parameters that need to be calibrated concern the PACS event-points present in the NetLogo world. These points are colored with different shades of magenta (see Fig.S1), one for each of the 10 PACS classes, and randomly move around the world during a simulation run with a frequency much greater than the simulation time step, that in our model corresponds to 1 year (in particular, each point shifts of 2 patches towards a random direction 73 times during each time step t - i.e. with a frequency equivalent to 5 days).

As explained in the MP, in our model the PACS event-points represent opportunities, ideas, encounters, intuitions, serendipity events, etc., which can periodically, and randomly, occur to a given researcher along her career. The relative abundance of points belonging to each PACS class is fixed in agreement with the information of the APS data set and it can be appreciated in the histogram shown in the left panel of Fig.S3 (for example, it appears that the PACS code 70 is the most expressed, while the PACS code 90 is the less present). The total number N_E of these points is one of the global parameters that have to be calibrated.

The dynamical rules of the model, presented in detail in the MP, assume that the N researchers, during their careers, are exposed to events and ideas which could trigger research lines, with the consequent articles production, along one or more different disciplinary fields according with the PACS numbers associated to each of the N_E event-points. A given researcher A_i, depending on her interdisciplinary index I_i^{APS}, is sensitive only to the points corresponding to the numbers present in her PACS array $\vec{D}_i^{APS}$; let us define these points as 'special' for that researcher. Every year t, a check is performed over all the researchers in order to verify what and how many event-points would fall inside their "sensitivity circles", which represent the extension of their sensitivity to the special points and therefore influence the publication dynamics. In the right panel of Fig.S3 is shown a zoom of the world, where three researchers, belonging to the three interdisciplinarity groups L_1^{APS}, L_2^{APS} and L_3^{APS}, are reported together with their "sensitivity circles". The sizes of these circles are other three parameters that have to be calibrated through the comparison with real data.

In the left panel of Fig.S4 we show an example of the publication dynamics for the generic author A_i. Let us suppose that $0 < D_i(t) \leq D_i^{APS}$ is the number of special PACS points randomly falling in the sensitivity circle of A_i at time t. In this example $D_i^{APS} = 4$ but $D_i(t) = 3$ since, among the four PACS numbers (1, 4, 6, 8) present in the array $\vec{D}_i^{APS}$ (real data), only three (1, 4, 8) do fall inside the circle. We can therefore define a temporary array $\vec{D}_i(t)$ containing these numbers. At this point, as explained in the MP, the considered researcher compares its talent T_i with a random real number $r \in [0,1]$. Let us suppose that $r < T_i$: in this case the number $P_i(t)$ of her published papers increases of an integer quantity $\Delta P_i(t)$ randomly extracted from a Normal distribution with mean $m_{P_i}(t-1) = \mu P_i(t-1)$ and standard deviation $\sigma_{P_i}(t-1) = \gamma P_i(t-1)$. The factors μ and γ are other two global parameters (both ≤ 1) that have to be determined by the comparison with real data (notice that these parameters

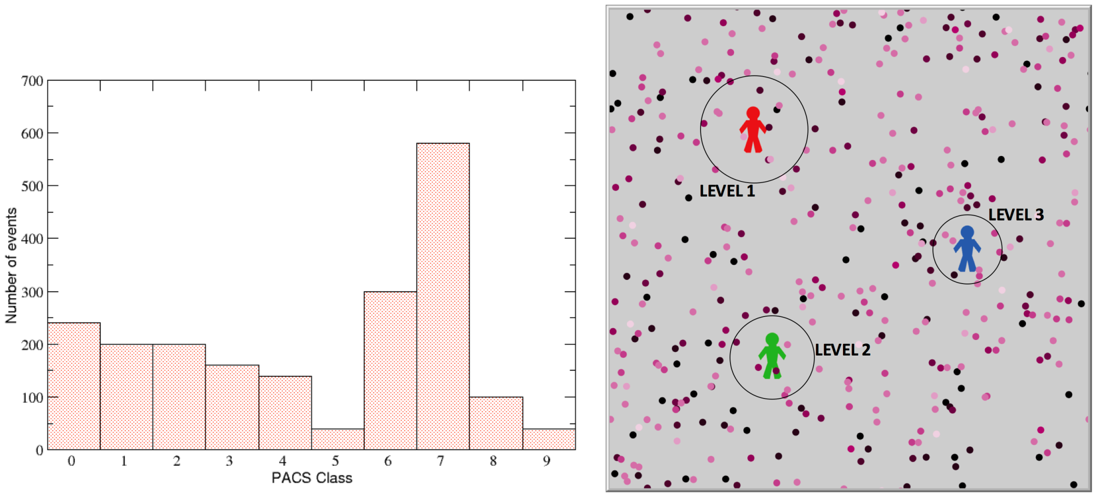

FIG. S3: Left panel: A histogram showing the number of event points for each PACS class, over a total of $N_E = 2000$, according to its relative percentage abundance in the APS dataset. Right panel: A zoom from Fig.S1, where only three researchers, each belonging to one of the three interdisciplinarity levels, are reported with their colors: red (level 1), green (level 2) and blue (level 3). Around them, some moving events are visible, represented as points of different colors selected from a magenta scale. Each color corresponds to a given PACS class of the APS data set, numbered from 0 (darkest) to 9 (brightest), as also shown in Fig.S1. The relative percentage of event points of each class is different and corresponds to the real one. Around each of the three researchers, the corresponding sensitivity circle is also visible, whose radius decreases by increasing the interdisciplinarity level (see text).

are fixed in time and are common to all the authors, while $m_{P_i}(t-1)$ and $\sigma_{P_i}(t-1)$ are different for each author A_i and are also variable in time, since they do depend on her past production at time $t-1$). Finally, all the new ΔP_i publications will be characterized by the PACS numbers contained in the array $\vec{D}_i(t)$. In the example of Fig.S4 $\Delta P_i = 3$, thus three new papers will be added to the papers array $\vec{P}_i(t-1)$ obtaining the new updated array $\vec{P}_i(t)$ where each of the new papers is characterized by the same three PACS numbers (1, 4, 8) – in practice, for each paper a copy of the array $\vec{D}_i(t)$ is saved.

The rationale behind these rules is twofold. On one hand, each researcher A_i exploits the opportunities offered by the event-points falling in her sensitivity circle with a probability proportional to her talent, i.e. more talented authors have a greater a-priori probability of publishing new papers. On the other hand, the periodic increment in the number of publications is a constant fraction of the already published papers, i.e. the greater is the number $P_i(t-1)$ of existing publications at time $(t-1)$, the higher is the number ΔP_i of new publications at time t (a sort of Matthew effect). Of course several approximations with respect to the reality have been assumed here. In particular, we assign the same PACS numbers to all the new papers published by A_i at time t and we do not consider coauthoring in the papers publication (each paper has a single author). This latter approximation contributes to produce an excess of published papers at the end of a simulation, but this is not a problem since we are interested in reproducing only the stylized fact represented by the shape of the papers distribution.

In order to choose the correct values for the global parameters previously introduced, i.e. the total number N_E of event-points, the radius of the sensitivity circles and the factors μ and γ, we have run several simulation tests with different combinations of these parameters and compared the numerical results with the real APS data.

First, we considered the averages $< d_i^{sim} >_g$, calculated over all the authors of the three groups $(g = 1, 2, 3)$, of the average number $d_i^{sim}(t_{max})$ of different PACS simultaneously present in their publications at the end of the simulation (i.e. at $t = t_{max}$) and compared them with the analogous real values $< d_i^{APS} >_g$ $(g = 1, 2, 3)$. It turned out that the values of $< d_i^{sim} >_g$ strictly depend on both the total number N_E of event-points and the radius of the sensitivity circles. The choice of $N_E = 2000$ and of a radius of 6.5, 5.2 and 4.9 patches for the groups L_1,L_2 and L_3 respectively, was able to produce the best agreement with the APS data, with an error of 1%, as shown in the right panel of Fig.S4. The decreasing size of the radius of the sensitivity circles for increasing interdisciplinarity levels, can be also justified by the evidence that the probability for a given researcher A_i to find special event-points inside her sensitivity circle increases with D_i^{APS}, and therefore with the interdisciplinarity index I_i^{APS}, thus if we adopted the same size of the circles for the three groups L_1, L_2 and L_3, we would introduce a bias in favor of authors with medium

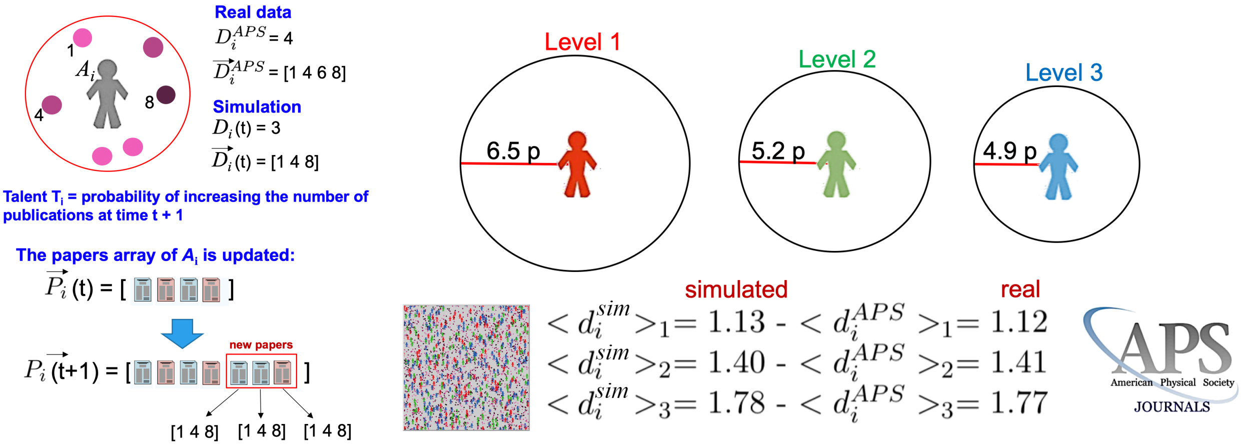

FIG. S4: (Left panel) An example of the dynamical rules for the publication of papers, see text. (Right panel) The total number N_E of event-points and the radius of the sensitivity circles for the three groups of authors can be chosen by looking at the agreement between the average values of the simulated $d_i(t_{max})$ and the corresponding d_i^{APS} obtained from the APS data set, see text.

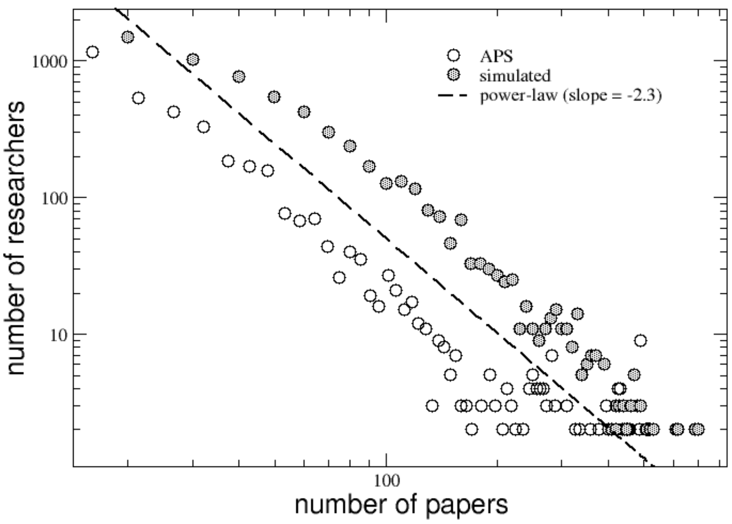

FIG. S5: Comparison between the papers distribution obtained from APS data set (open circles) and that obtained with the model simulation with $N_E = 2000$, $\mu = 1/5$ and $\gamma = 1/4$ (full circles). The two distributions show a power-law behavior with the same exponent -2.3.

and in particular with high interdisciplinarity level.

Second, we were able to choose the correct values for the factors μ and γ by comparing the simulated distribution of all the published papers (without distinctions among the interdisciplinarity levels) with the real one extracted from the APS data set. It turned out that the choice $\mu = 1/5$ and $\gamma = 1/4$ was able to produce a simulated papers distribution with a power-law behavior with the same slope (-2.3) of the real one (see Fig.S5). Notice that, due to the constraints imposed by the calibration, these first results are very robust and do not depend on the details of the initial conditions of the simulations (i.e. do not depend neither on the particular realization of the distribution of talent among the agents, nor on the initial random position of both the agents and the event-points).

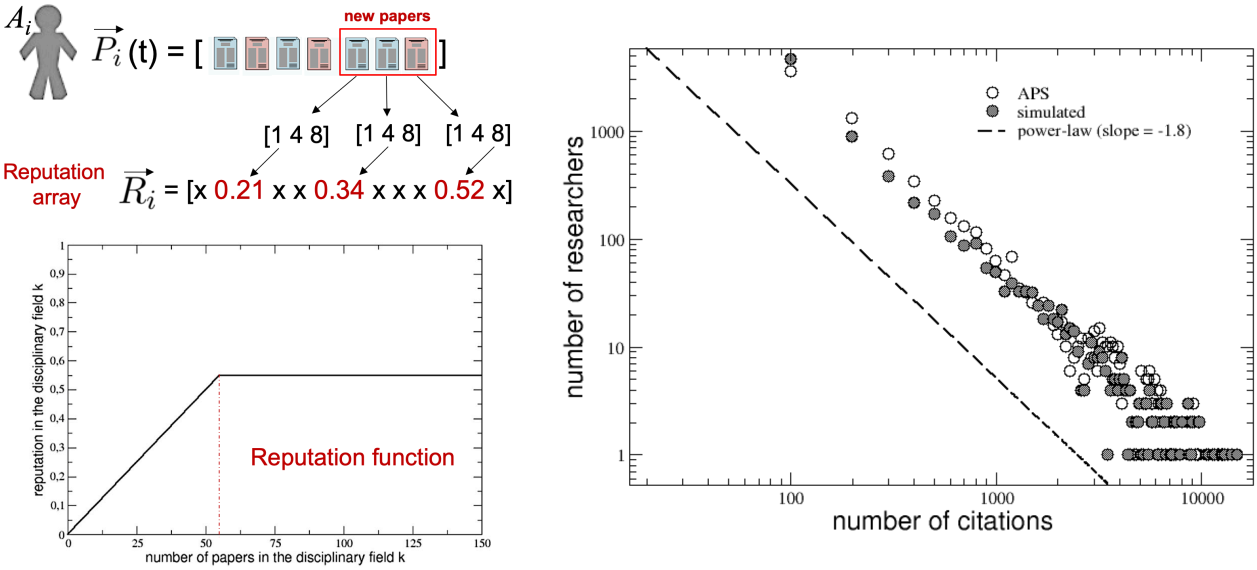

FIG. S6: (Left Panel) An example of the dynamical rules regulating the increase of reputation of an author A_i in the fields corresponding to the PACS present in each new publication, see text. (Right Panel) Comparison between the citations distribution obtained from APS data set (open circles) and that obtained with the model simulation with $k = 0.01$ and $y_{max} = 0.55$ (full circles). The two distributions show a power-law behavior with the same exponent -1.8.

Let us finally address the calibration of the citation dynamics for our model. As we have just seen, every year t all the researchers have the chance to increase their number of publications. In correspondence of each new paper, author A_i also increases her own reputation in each of the disciplinary fields indicated by the PACS numbers associated to that paper. As explained in the MP, each one of the 10 elements of the reputation array $\vec{R}_i(t)$ is a real number, included in the interval $[0, 1]$, representing the reputation level reached by the researcher A_i in the corresponding disciplinary field at time t (see the top-left panel of Fig.S6 for an example).

A plausible approximation to account the behavior of the reputation level y of a generic author in a given field at time t can be that of considering it as a semi-linear function of the number x of papers published in that field at time t. In other words, we assume that y does vary with x following the function

$$y = \begin{cases} k \cdot x & for \quad x < x_{th} \\ y_{max} & for \quad x \geq x_{th} \end{cases}$$

where k and y_{max} are global parameters that, again, have to be calibrated with the real data, while x_{th} is the abscissa of the inflection point (that depends on y_{max}).

Since, following the publication/citation dynamical rules explained in the MP, the total number of citations $C_i(t+1)$ reached by the author A_i at time $t + 1$ does depend on both her citation score $C_i(t)$ and her reputation array $\vec{R}_i(t)$ at time t (Matthew effect), the choice of k and y_{max} does influence the citations distribution obtained at the end of a simulation (i.e. at $t = t_{max}$). Through several simulation tests, where different combinations of these parameters were adopted, we found that the values $k = 0.01$ and $y_{max} = 0.55$ (see bottom-left panel of Fig.S6) were able to produce a simulated overall citations distribution (without distinctions among the interdisciplinarity levels) that overlaps the analogous distribution obtained from the APS data set, following a power-law behavior with the same slope (-1.8, see the right panel of Fig.S6). Again, the constraints imposed by the comparison with the real data make these simulation results very robust, substantially independent from the initial conditions.

In conclusion, as last point to address, we also notice that - as observed in the MP - the calibrated agreement between the simulated averages $< d_i^{sim} >_g$ and the analogous real ones $< d_i^{APS} >_g$ for the three interdisciplinarity groups ($g = 1, 2, 3$) do not ensure, of course, the correspondence of the individual $d_i^{sim}(t_{max})$ ($i = 1, ..., N$) of each agent-author at the end of a simulation with her initially assigned d_i^{APS}. Being the D_i^{APS} fixed for all the authors

during the simulation, this also implies that their initial value of the (real) individual interdisciplinarity index I_i^{APS} can be different with respect to the corresponding one $I_i^{sim}(t_{max}) = D_i^{APS} \times d_i^{sim}(t_{max})$ obtained at the end of the simulation. As a consequence, after a given simulation run, all the authors have to be reassigned – on the basis of the same rules described in paragraph 1.1 – to the three interdisciplinarity groups before calculating the corresponding papers and citations distributions (as those showed in the MP). We call these new groups L_1^{sim}, L_2^{sim} and L_3^{sim}. It results that the number of authors belonging to L_1^{sim}, L_2^{sim} and L_3^{sim} is not exactly the same of the number of authors belonging to the original groups L_1^{APS}, L_2^{APS} and L_3^{APS}, but typically the differences between the old and the new groups do not exceed 10%. In the simulation results presented in the MP, the sizes of the three new groups were, respectively, $N_1 = 2591$, $N_2 = 2383$ and $N_3 = 2329$. With respect to the original sizes shown in Table S1 of Appendix 1, we notice that L_1^{sim} slightly increased the number of its members, group L_2^{sim} slightly decreased it, while group L_3^{sim} leaved it relatively unchanged.

[1] Wilensky, U. (1999). NetLogo. http://ccl.northwestern.edu/netlogo/. Center for Connected Learning and Computer-Based Modeling, Northwestern University, Evanston, IL.
